# Cytosolic serpins act in a cytoprotective feedback loop that limits ESX-1-dependent death of *Mycobacterium marinum*-infected macrophages

**DOI:** 10.1128/mbio.00384-24

**Published:** 2024-08-01

**Authors:** Esther Nobs, Katie Laschanzky, Kristina Munke, Elin Movert, Christine Valfridsson, Fredric Carlsson

**Affiliations:** 1 Department of Biology, Lund University, Lund, Sweden; 2 Department of Experimental Medical Science, Lund University, Lund, Sweden; Washington University School of Medicine, St. Louis, Missouri, USA; University of Notre Dame, Notre Dame, Indiana, USA

**Keywords:** bacterial pathogenesis, host cell death, membrane permeabilization, cytosolic surveillance pathways, type VII secretion system, host-pathogen interactions, type I interferon, Spi2A, serpins, cathepsin B, lysosome

## Abstract

Serine protease inhibitors (serpins) constitute the largest family of protease inhibitors expressed in humans, but their role in infection remains largely unexplored. In infected macrophages, the mycobacterial ESX-1 type VII secretion system permeabilizes internal host membranes and causes leakage into the cytosol of host DNA, which induces type I interferon (IFN) production via the cyclic GMP-AMP synthase (cGAS) and stimulator of IFN genes (STING) surveillance pathway, and promotes infection *in vivo*. Using the *Mycobacterium marinum* infection model, we show that ESX-1-mediated type I IFN signaling in macrophages selectively induces the expression of *serpina3f* and *serpina3g*, two cytosolic serpins of the clade A3. The membranolytic activity of ESX-1 also caused leakage of cathepsin B into the cytosol where it promoted cell death, suggesting that the induction of type I IFN comes at the cost of lysosomal rupture and toxicity. However, the production of cytosolic serpins suppressed the protease activity of cathepsin B in this compartment and thus limited cell death, a function that was associated with increased bacterial growth in infected mice. These results suggest that cytosolic serpins act in a type I IFN-dependent cytoprotective feedback loop to counteract the inevitable toxic effect of ESX-1-mediated host membrane rupture.

**IMPORTANCE:**

The ESX-1 type VII secretion system is a key virulence determinant of pathogenic mycobacteria. The ability to permeabilize host cell membranes is critical for several ESX-1-dependent virulence traits, including phagosomal escape and induction of the type I interferon (IFN) response. We find that it comes at the cost of lysosomal leakage and subsequent host cell death. However, our results suggest that ESX-1-mediated type I IFN signaling selectively upregulates *serpina3f* and *serpina3g* and that these cytosolic serpins limit cell death caused by cathepsin B that has leaked into the cytosol, a function that is associated with increased bacterial growth *in vivo*. The ability to rupture host membranes is widespread among bacterial pathogens, and it will be of interest to evaluate the role of cytosolic serpins and this type I IFN-dependent cytoprotective feedback loop in the context of human infection.

## INTRODUCTION

The mycobacterium genus harbors ubiquitous environmental species as well as major human pathogens. *Mycobacterium tuberculosis* and its closely related model organism *Mycobacterium marinum* (1) have intracellular lifestyles and reside primarily in neutrophils and CD64^+^ cells of myeloid origin *in vivo* (2–4). The functionally conserved ESX-1 type VII secretion system is required for the virulence of both species (5–8). ESX-1 interacts with infected macrophages to promote intracellular bacterial replication (5, 6, 9), and it is required for activation of the inflammasome (8, 10–12) and the cyclic GMP-AMP synthase (cGAS) and stimulator of interferon genes (STING) pathway (12–18). Activation of these cytosolic surveillance pathways depends on ESX-1-mediated permeabilization of internal host membranes, where the release of host DNA into the cytosol is sensed by cGAS to induce the production of type I interferon (IFN) (12, 18, 19).

Active tuberculosis is associated with a robust type I IFN transcriptional profile (20), and analyses in mice have demonstrated that the type I IFN response promotes bacterial growth and disease development *in vivo* (21–24). Signaling via the type I IFN receptor leads to the expression of many different interferon-stimulated genes (ISGs) with diverse functions (14, 25). Large-scale transcriptional screens suggest that so-called clade A3 serine protease inhibitors (serpins) may be induced by infection with *M. tuberculosis* (22) and *Mycobacterium bovis* BCG (26), but the regulation and role of serpins in infection remain largely unexplored.

Serpins constitute a large superfamily of proteins that inhibit serine proteases, and there are also examples of serpins that inhibit caspases and cysteine proteases (27, 28). Using the *M. marinum* infection model, we here report that ESX-1-mediated type I IFN signaling selectively induces the production of cytosolic clade A3 serpins (*serpina3f* and *serpina3g*). We find that these serpins act in a type I IFN-dependent feedback loop to limit ESX-1-mediated cell death of infected macrophages—a cytoprotective function associated with increased bacterial growth *in vivo*.

## RESULTS

### ESX-1 induces type I IFN-dependent expression of cytosolic A3 serpins

**Fig 1:**
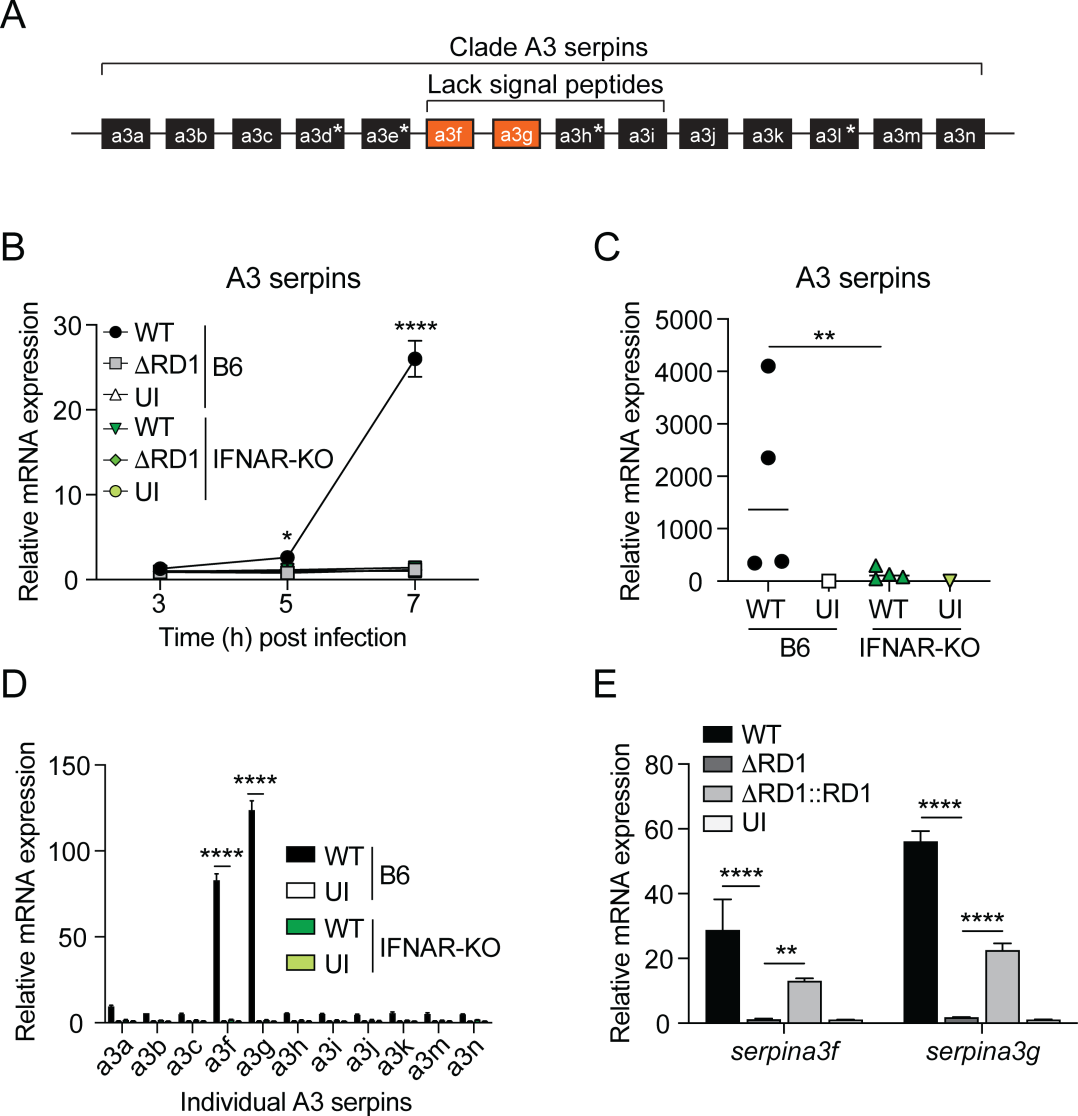
ESX-1 induces type I IFN-dependent expression of cytosolic A3 serpins. (**A**) Schematic representation of the genetic region encoding A3 serpins in mice. Pseudogenes, not encoding a functional protein, are marked with an asterisk (30, 31), and *serpina3f/g* are highlighted in orange. *serpina3f–i* do not encode a signal peptide (30). (**B, D, and E**) Wild-type C57BL/6 (B6) and IFNAR-KO macrophages were infected with WT, ΔRD1, and ΔRD1::RD1 *M. marinum* at a multiplicity of infection (MOI) of 5, or left uninfected (UI), as indicated. (**B**) Kinetic reverse-transcription quantitative PCR (RT-qPCR) analysis gene expression, using primers against regions conserved among clade A3 serpin encoding genes. (**C**) B6 and IFNAR-KO mice were infected with WT *M. marinum*. At 14 days post-infection, the infected tail tissue was analyzed by RT-qPCR for the expression of A3 serpins as described for panel B above. Shown is the mean of four infected mice (and one uninfected mouse) per genotype, as indicated. Two-tailed unpaired *t*-test, **P < 0.01. (**D**) Expression of the individual A3 serpin encoding genes at 7 hpi. (**E**) RT-qPCR analysis of *serpina3f* and *serpina3g* expression at 7 hpi. Results (mean ± standard deviation [SD]; *n* = 3) are representative of three independent experiments. One-way analysis of variance (ANOVA), ****P < 0.0001. (**B and D**) Results (mean ± SD; *n* = 3) are representative of three independent experiments. Two-way ANOVA, ****P < 0.0001.

To explore the effect of ESX-1-mediated induction of type I IFN on the production of clade A3 serpins in mice, we devised primers complementary to genetic regions with a high level of conservation among all A3 serpins (Fig. 1A; Table S1). Expression analysis in C57BL/6 (B6) bone marrow-derived macrophages infected with wild-type (WT) *M. marinum* or an isogenic ESX-1-deficient mutant (ΔRD1) indicated that *M. marinum* induces the expression of A3 serpins in an ESX-dependent manner (Fig. 1B). Analysis of type I IFN receptor 1-deficient (IFNAR-KO) macrophages demonstrated that the expression of A3 serpins was completely dependent on type I IFN signaling (Fig. 1B). The requirement for type I IFN signaling translated into the infected tissue in a mouse model of *M. marinum* infection (Fig. 1C), where the bacteria grow and cause granulomatous disease selectively in tail tissue due to the low optimal growth temperature of the bacteria and the cooler environment in the tail (8). To investigate the expression of the individual A3 serpins in macrophages, we generated primers against gene-specific sequences in their active sites (Fig. 1A; Table S1). This more detailed analysis revealed that ESX-1-mediated type I IFN signaling selectively induced the expression of *serpina3f* and *serpina3g* (Fig. 1D), two serpins that lack signal peptides and localize to the cytosolic compartment (Fig. 1A) (29, 30). Expression of *serpina3f* and *serpina3g* was maintained at 24 hours post-infection (hpi), albeit at a much lower level (Fig. S1). Complementation of the ΔRD1 strain with the RD1-region from *M. tuberculosis* (ΔRD1::RD1) restored the ability to induce the expression of *serpina3f* and *serpina3g* (Fig. 1E). Collectively, these results suggest that ESX-1-dependent type I IFN signaling selectively induces the expression of cytosolic serpins in mycobacteria infected macrophages.

### Cytosolic serpins inhibit the activity of extralysosomal cathepsin B

**Fig 2:**
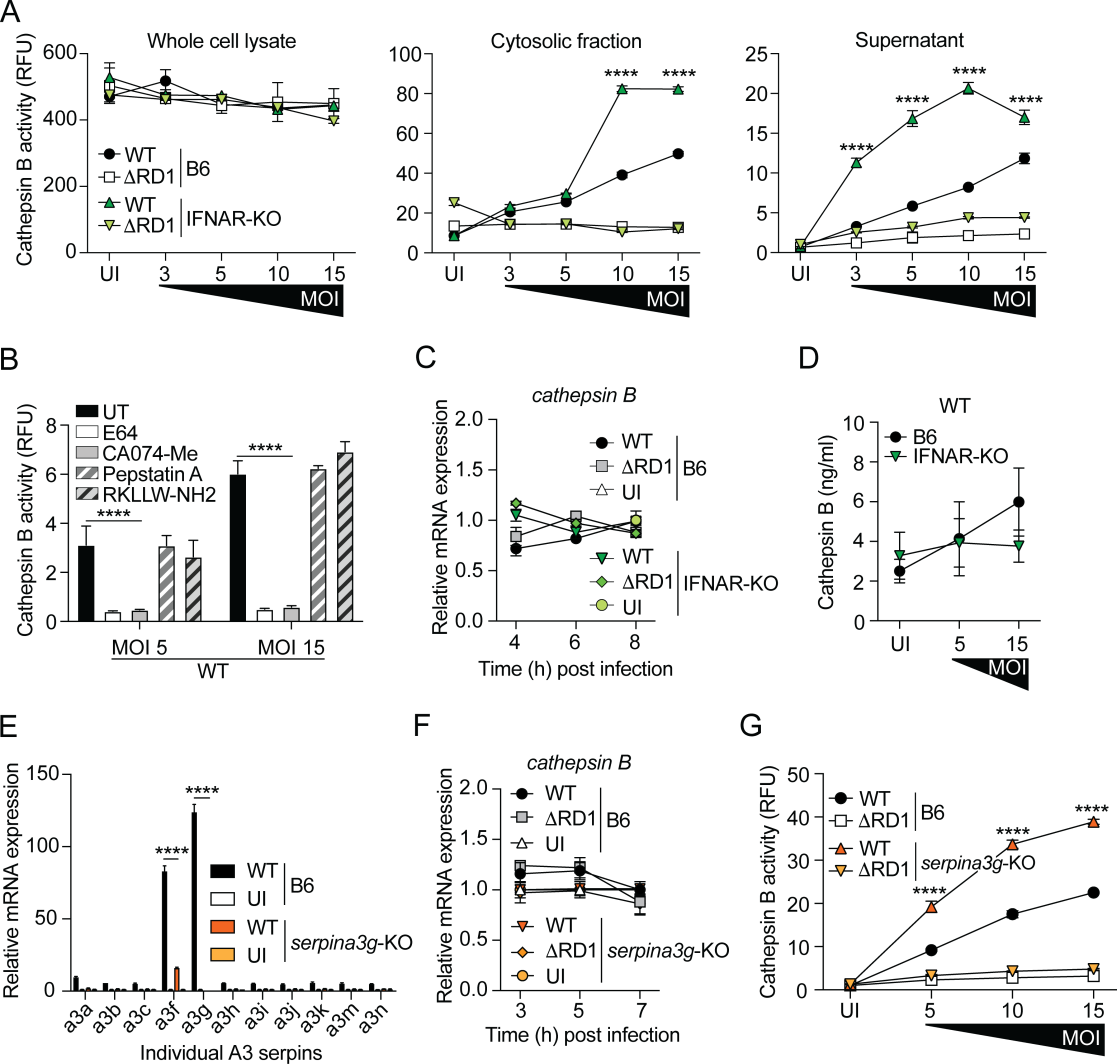
Cytosolic serpins inhibit the activity of extralysosomal cathepsin B. Macrophages were infected with WT and ΔRD1 *M. marinum*, or uninfected (UI), as indicated. (**A**) At 7 hpi, cathepsin B activity was measured in whole cell lysate, cytosolic fraction, and supernatant, as indicated. (**B**) Analysis of cathepsin B activity in the supernatant of WT-infected macrophages treated with inhibitors of cathepsin B (CA074-Me; 25 µM), cysteine proteases (E64; 10 µM), aspartyl proteases (pepstatin A; 10 µM). or cathepsin L (RKLLW-NH2; 10 µM). Untreated (UT) supernatants were analyzed as controls. (**C**) Kinetic RT-qPCR analysis of *cathepsin B* expression in macrophages infected (MOI = 5) as indicated. (**D**) Enzyme-linked immunosorbent assay-based analysis of cathepsin B protein concentration in the cytosolic fraction of WT-infected B6 and IFNAR-KO macrophages. Results (mean ± SD; *n* = 4) are representative of two independent experiments. Two-way ANOVA. (**E**) RT-qPCR analysis of the expression of the individual A3 serpin encoding genes in WT-infected B6 and *serpina3g*-KO macrophages (MOI = 5) at 7 hpi, as indicated. Uninfected (UI) cells were analyzed as controls. (**F**) Kinetic RT-qPCR analysis of *cathepsin B* expression in macrophages infected (MOI = 5) as indicated. (**G**) Analysis of cathepsin B activity in the supernatant of WT-infected B6 and *serpina3g*-KO macrophages, as indicated. (**A–C and E–G**) Results (mean ± SD; *n* = 3) are representative of three independent experiments. Two-way ANOVA, ****P < 0.0001.

Previous studies have suggested that Spi2A, the protein encoded by *serpina3g*, inhibits the activity of cathepsin B that has leaked into the cytosol from the lysosomal compartment (32–39). To investigate cathepsin B activity in the cytosol of infected macrophages, we prepared three different fractions of cells infected with titrated amounts of WT or ΔRD1 *M. marinum*: whole cell lysate, the cytosolic fraction, and the supernatant (Fig. 2A). Analysis of whole cell lysates demonstrated similar levels of total cathepsin B activity in all conditions, including the uninfected control (Fig. 2A, left panel). However, only infection with ESX-1-proficient *M. marinum* produced cathepsin B activity in the cytosolic compartment (Fig. 2A, middle panel), suggesting that ESX-1-dependent permeabilization of the lysosomal membrane causes leakage of cathepsin B into the cytosol. IFNAR-KO macrophages infected with WT bacteria exhibited significantly higher cytosolic cathepsin B activity compared to similarly infected B6 cells (Fig. 2A, middle panel), suggesting that the type I IFN response suppresses the activity of extralysosomal cathepsin B. The cathepsin B activity in the supernatant principally mimicked the results obtained in the cytosolic fraction (Fig. 2A, middle and right panels), allowing us to use the more easily prepared supernatant as a proxy for the cytosolic fraction.

Extralysosomal cathepsin B activity in WT-infected macrophages was inhibited by the general cysteine protease inhibitor E64 and the cathepsin B inhibitor CA074-Me, but not by the aspartyl protease inhibitor pepstatin A or the cathepsin L specific inhibitor RKLLW-NH2 (Fig. 2B), suggesting that our assay measures the activity of cathepsin B specifically. Analysis at the transcriptional level demonstrated similar expression of *cathepsin B* in all experimental conditions (Fig. 2C), and the leakage of cathepsin B protein into the cytosolic compartment in WT-infected IFNAR-KO macrophages was not increased as compared to similarly infected B6 cells (Fig. 2D). Thus, our results suggest that type I IFN signaling does not inhibit the production or cytosolic translocation of cathepsin B, but specifically regulates the activity of extralysosomal cathepsin B (Fig. 2A through D).

To determine the role of cytosolic serpins in type I IFN signaling-dependent suppression of extralysosomal cathepsin B activity in infected cells, we generated macrophages from *serpina3g*-KO mice. Analysis of the individual A3 serpins confirmed that expression of *serpina3g* was abolished in infected *serpina3g*-deficient macrophages (Fig. 2E). The expression of the adjacent *serpina3f* gene was also diminished in *serpina3g*-KO cells (Fig. 2E), allowing us to use this mouse model to investigate the role of both of the cytosolic serpins regulated by ESX-1-mediated type I IFN signaling (Fig. 1D). The lack of cytosolic serpins in *serpina3g*-KO macrophages did not significantly affect the production of type I IFN in response to infection (Fig. S2), and similar to the situation in IFNAR-KO macrophages (Fig. 2C), the production of cathepsin B was unaffected in *serpina3g*-KO cells (Fig. 2F). Importantly, the activity of extralysosomal cathepsin B in WT *M. marinum* infection was significantly increased in *serpina3g*-KO cells compared to B6 macrophages (Fig. 2G), indicating that the serpins are indeed required to inhibit the enzymatic activity of cathepsin B that has leaked into the cytosol from the lysosomal compartment.

### Cytosolic serpins do not regulate inflammasome activation in *M. marinum*-infected macrophages

**Fig 3:**
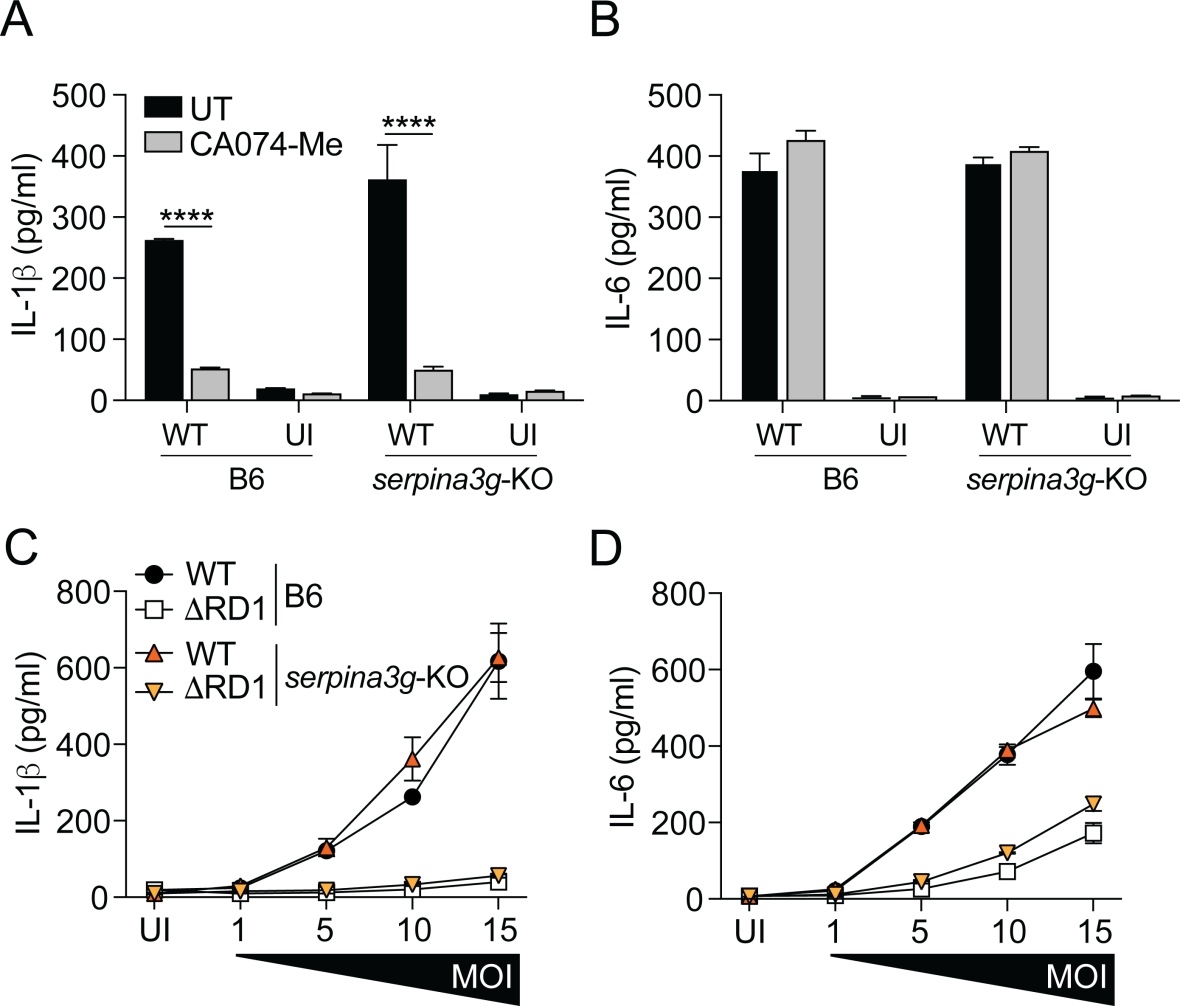
Cytosolic serpins do not regulate inflammasome activation in *M. marinum*-infected macrophages. (**A and B**) B6 and *serpina3g*-KO macrophagesmacrophages were infected with WT *M. marinum* (MOI = 10) and treated with the cathepsin B inhibitor CA074-Me (25 µM), as indicated. The concentration of IL-1β (**A**) and IL-6 (**B**) secreted into the supernatant was determined by enzyme-linked immunosorbent assay (ELISA) at 24 hpi. (**C and D**) B6 and *serpina3g*-KO macrophages were infected with WT and ΔRD1 *M. marinum* at titrated MOI, as indicated. The concentration of IL-1β (**C**) and IL-6 (**D**) secreted into the supernatant was determined by ELISA at 24 hpi. (**A–D**) Results (mean ± SD; *n* = 3) are representative of three independent experiments (two-way ANOVA, ****P < 0.0001).

It is well established that *M. tuberculosis* and *M. marinum* activate the inflammasome in an ESX-1-dependent manner (8, 10–12). Previous studies with *M. tuberculosis* and *M. marinum* have implicated the release of cathepsin B into the cytosol of infected macrophages in this process, which was inhibited by the cathepsin B inhibitor CA074-Me (40, 41). This finding prompted us to explore if the type I IFN-induced cytosolic serpins play a role in regulating inflammasome activation in infected macrophages. To this end, we measured the secretion of interleukin-1β (IL-1β) from B6 and *serpina3g*-KO macrophages infected with WT *M. marinum* in the presence or absence of CA074-Me. While addition of CA074-Me prevented the secretion of IL-1β (Fig. 3A), *serpina3g*-KO macrophages exhibited no significant phenotype (Fig. 3A), suggesting that the induction of cytosolic serpins does not regulate inflammasome activation. The secretion of IL-6, which occurs independently of the inflammasome, was analyzed as a control (Fig. 3B). Analysis over a range of multiplicities of infection further established that the cytosolic serpins do not regulate ESX-1-mediated inflammasome activation in macrophages (Fig. 3C and D).

### Cytosolic serpins inhibit ESX-1-dependent host cell death

**Fig 4:**
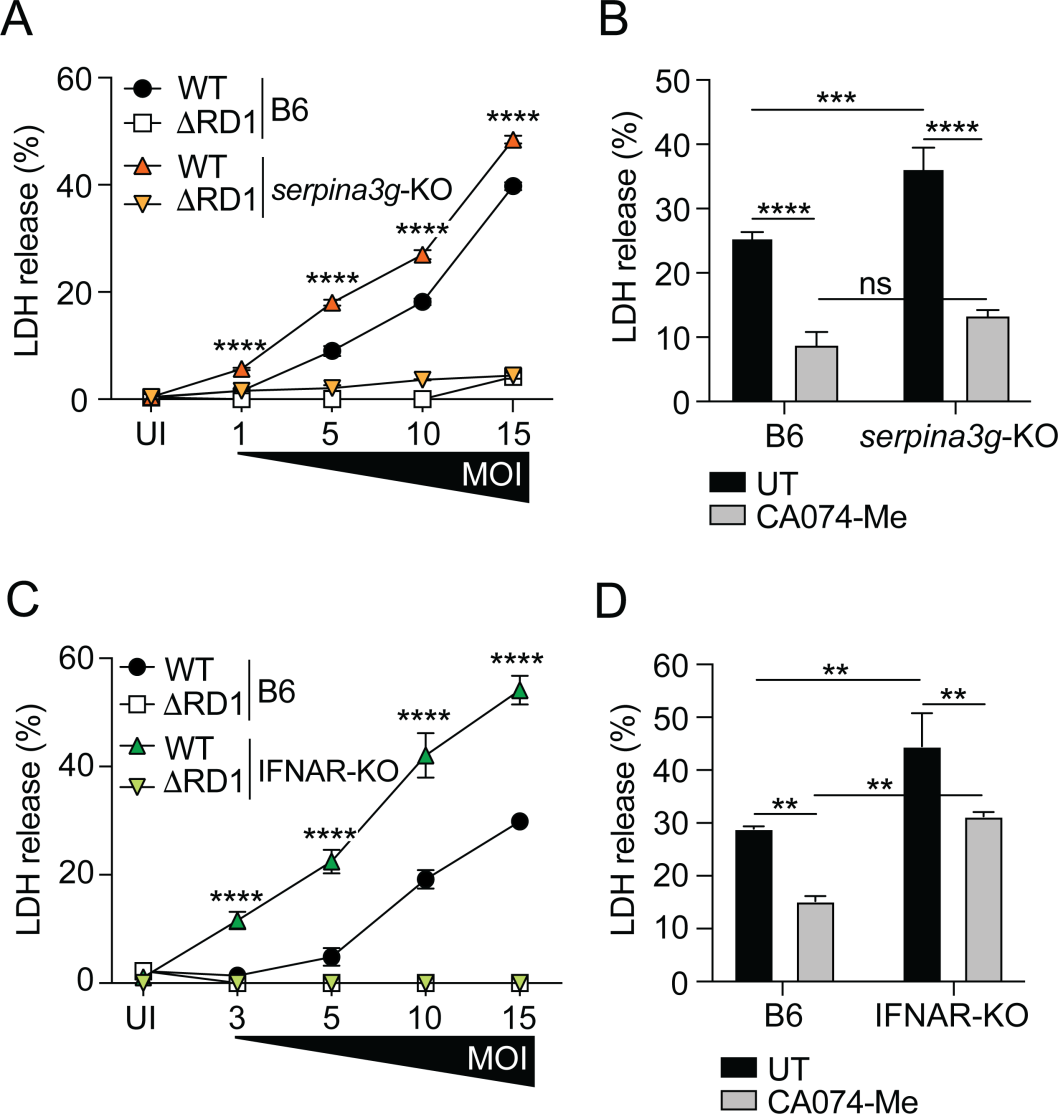
Cytosolic serpins inhibit ESX-1-dependent host cell death. B6, IFNAR-KO, and *serpina3g*-KO macrophages were infected with WT and ΔRD1 *M. marinum*, or uninfected (UI), as indicated. (**A**) LDH in the supernatant of B6 and *serpina3g*-KO macrophages infected at increasing MOI was measured at 7 hpi. (**B**) WT-infected macrophages (MOI = 10) were treated with the cathepsin B inhibitor CA074-Me (25 µM), and LDH release into the supernatant was measured at 7 hpi. (**C**) LDH in the supernatant of B6 and IFNAR-KO macrophages infected at increasing MOI was measured at 7 hpi. (**D**) WT-infected macrophages (MOI = 10) were treated with CA074-Me (25 µM), and LDH release into the supernatant was measured at 7 hpi. (**A–D**) Results (mean ± SD; *n* = 3) are representative of three independent experiments. Two-way ANOVA, ****P < 0.0001.

The membranolytic activity of the ESX-1 secretion system leads to several types of necrotic host cell death (42), suggesting a high degree of redundancy between different cell death pathways in mycobacterial infection. The leakage of cathepsin proteases from permeabilized lysosomes can cause lethal cellular autodigestion (40, 43), where cathepsin B has been described as a key protease due to its ability to function at cytosolic pH (44). To explore the role of cytosolic serpins in regulating ESX-1-dependent cytotoxicity, we infected B6 and *serpina3g*-KO macrophages with titrated amounts of WT or ΔRD1 *M. marinum* and analyzed cell death by the release of lactate dehydrogenase (LDH) into the supernatant at 7 hpi—an optimal time point to detect cathepsin B-dependent cytotoxicity in synchronized cell cultures (32, 33). As expected, *M. marinum* induced ESX-1-dependent cell death (Fig. 4A). The cell death caused by WT bacteria was significantly increased in *serpina3g*-KO compared to B6 macrophages (Fig. 4A), demonstrating that the cytosolic serpins limit ESX-1-dependent cell death. The protective effect of the serpins was no longer discernible at 24 hpi (Fig. S3A), a feature likely explained by the aforementioned redundancy between different cell death pathways and/or by the reduced expression of *serpina3f* and *serpina3g* at this later time point (Fig. S1). This feature might also explain why intracellular growth of *M. marinum* was unaffected in *serpina3g*-KO macrophages over a 72-hour period *in vitro* (Fig. S4). The cell death observed in WT-infected macrophages at 7 hpi was significantly reduced in both B6 and *serpina3g*-KO macrophages treated with CA074-Me (Fig. 4B), supporting the interpretation that the serpins protect against cell death induced by ESX-1-dependent release of cathepsin B into the cytosol.

Analysis of IFNAR-KO macrophages confirmed that type I IFN signaling was required for the suppression of ESX-1-dependent cell death (Fig. 4C; Fig. S3B). The relative increase of cell death in IFNAR-deficient macrophages (Fig. 4C) appeared greater than the difference between similarly infected *serpina3g*-KO and B6 cells (Fig. 4A). Moreover, unlike the situation in WT-infected *serpina3g*-KO macrophages (Fig. 4B), the addition of CA074-Me to IFNAR-KO did not suppress the release of LDH to the same level as in B6 macrophages (Fig. 4D). These results suggest that type I IFN signaling might, in addition to the cytosolic serpins, induce other yet unidentified cytoprotective ISGs.

### Cytosolic serpins promote *M. marinum* growth *in vivo*

**Fig 5:**
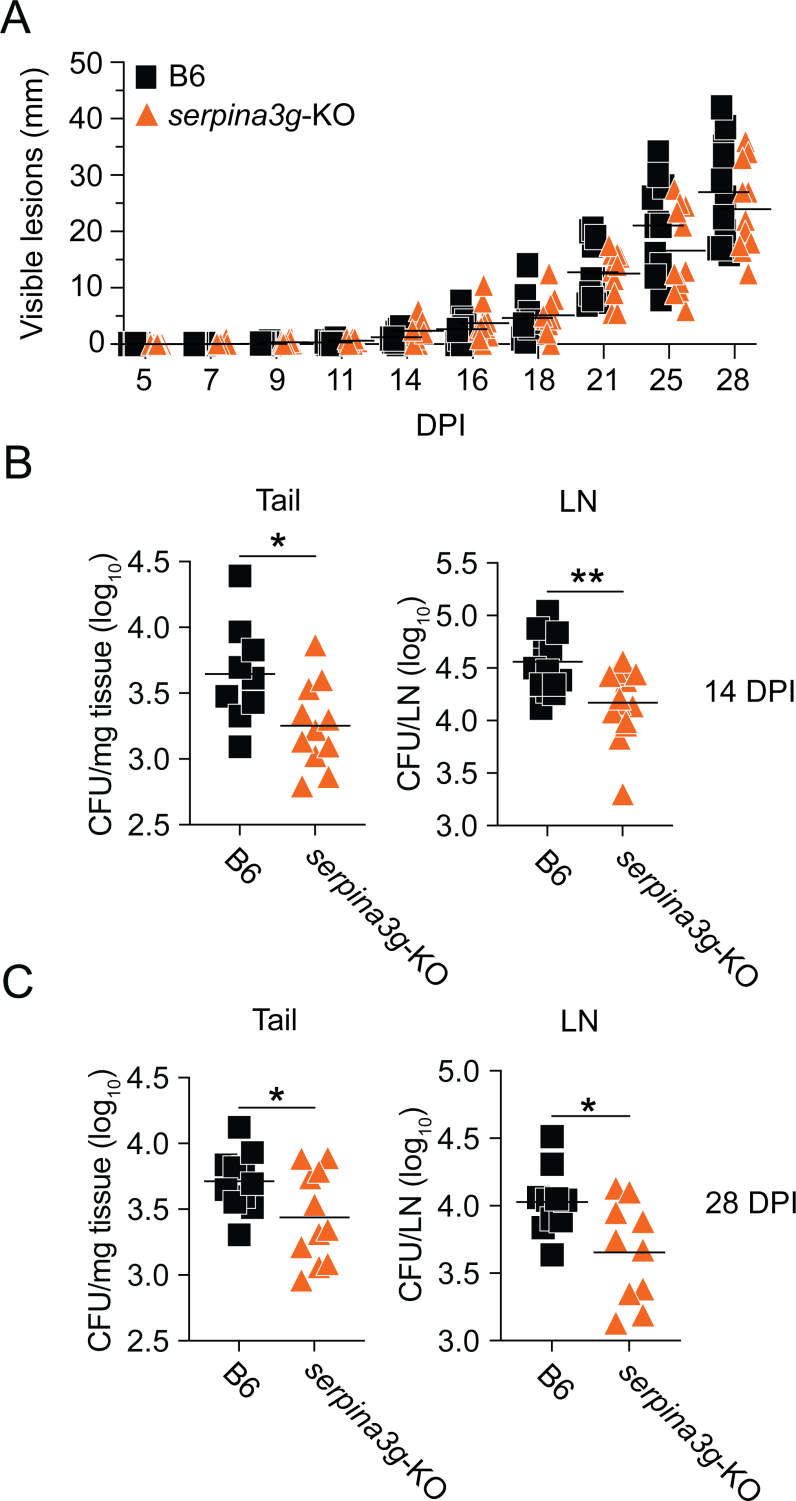
Cytosolic serpins promote *M. marinum* growth *in vivo*. B6 and *serpina3g*-KO mice were infected with WT *M. marinum* (1.4 × 10^7^ CFUs) via tail vein injection. (**A**) Kinetic analysis of the accumulated length (mm) of visible tail lesions. Each symbol indicates an individual mouse (*n* = 11 per group), and the bars show the mean for each group. Two-way ANOVA. (**B and C**) Bacterial burden in tail tissue and tail-draining (sciatic and inguinal, pooled) lymph nodes (LN) at 14 and 28 days post-infection (DPI), as indicated. Results (*n* = 9–15 mice per group) from two independent experiments. Bars indicate the mean for each group. Two-tailed unpaired *t*-test; *P < 0.05, **P < 0.01.

Because type I IFN signaling promotes disease development and mycobacterial growth *in vivo* (21–24), it was of interest to investigate whether the downstream induction of cytosolic serpins contributes to these effects. To this end, we infected B6 and *serpina3g*-KO mice with WT *M. marinum* and analyzed the development of visible tail lesions, the cellularity of the infected tissue, and bacterial growth. Kinetic analysis of the accumulated length of all visible lesions in individual tails suggested similar pathology in *serpina3g*-KO and B6 mice during the first 28 days of infection (Fig. 5A), and detailed flow cytometry-based analysis of the infected tissue indicated similar numbers of both neutrophils and monocyte-derived cells—regulating the level of inflammation in *M. marinum*-infected mice (3)—in both genotypes (Fig. S5). However, the determination of colony forming units (CFUs) in tail tissue and tail-draining lymph nodes at 14 and 28 days post-infection indicated significantly reduced bacterial growth in both tissues of *serpina3g*-KO as compared to B6 mice (Fig. 5B and C), demonstrating that the induction of cytosolic serpins augments mycobacterial growth *in vivo*.

## DISCUSSION

**Fig 6:**
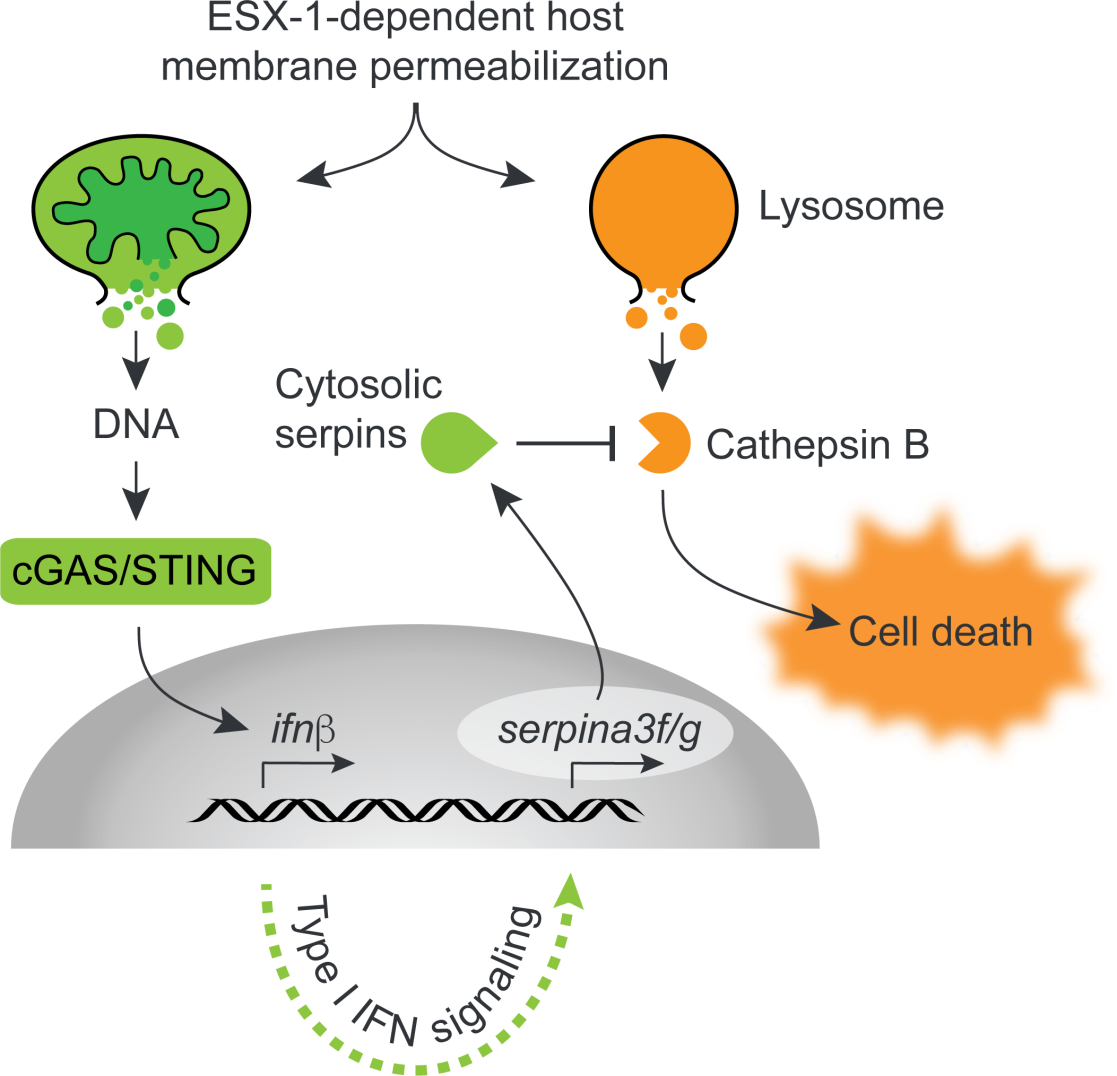
Working model. ESX-1-dependent permeabilization of host membranes causes leakage of host DNA (illustrated with a mitochondrion) into the cytosol and induces type I IFN via the cGAS-STING pathway. Type I IFN signaling leads to expression of the cytosolic serpins *serpina3f* and *serpina3g*, which inhibits cathepsin B as it reaches the cytosol due to ESX-1-dependent lysosomal rupture. Inhibition of cytosolic cathepsin B by serpins reduces the level of cell death *in vitro* and is associated with a higher bacterial burden *in vivo*.

It is well established that the membranolytic activity of the ESX-1 secretion system is required for necrotic cell death in infected macrophages (42), including ferroptosis (45), pyronecrosis (46), as well as Rip-kinase-dependent (47) and lysosome-dependent (40) cell death pathways. Of note, a recent study was unable to detect any of these types of cell death and instead reported a toxic effect of type I IFN signaling (48), a discrepancy that might be explained by differences in experimental conditions. Consistent with studies in *M. tuberculosis*-infected macrophages (41), our results indicate that ESX-1-dependent membrane permeabilization ruptures the lysosomal compartment and causes the release of cathepsin B into the cytosol. We find that this process promotes cell death of infected macrophages, but that concomitant type I IFN signaling selectively upregulates the production of cytosolic serpins and thereby limits ESX-1-dependent host cell death (Fig. 6). These results suggest that the type I IFN response—which, similar to cathepsin B leakage, is downstream of ESX-1-dependent host membrane permeabilization—initiates a protective feedback loop to limit the cytotoxic effects of cathepsin B leakage.

ESX-1-mediated disruption of host membranes leads to phagosomal escape of the bacteria (8, 40, 49, 50) and cytosolic release of DNA, which is sensed by the cGAS-STING pathway to induce type I IFN production in both *M. tuberculosis* and *M. marinum*-infected cells (12, 14, 18, 19). The type I IFN response is host-detrimental in mycobacterial infection (21–24), but it is not fully understood how type I IFN regulates the anti-mycobacterial immune response and promotes bacterial growth *in vivo*. Studies with *M. tuberculosis*, *Mycobacterium leprae*, and *M. marinum* suggest that this effect of type I IFN signaling may, at least in part, be explained by inhibition of IFNγ-mediated killing of the bacteria (23, 51, 52). The type I IFN response can also promote disease development by suppressing IL-1-dependent host protection (53–55). Our results add to the understanding of the functional role of the type I IFN response in mycobacterial infection and suggest that it may contribute to bacterial growth *in vivo* by limiting the demise of infected cells. We have previously shown that *M. marinum* resides primarily in neutrophils and monocyte-derived cells *in vivo*, and that monocytes protect against neutrophil-dependent inflammation in the infected tissue (3). Consistent with the similar pathology observed in B6 and *serpina3g*-KO mice, the lack of cytosolic serpins did not affect the cellularity of these cell types, which might be due to their high rates of turnover and phenotypic diversity of the infected tissue (3, 56). The dynamics of cellular turnover and phenotypic diversity *in vivo* might also explain why the effect of cytosolic serpins on bacterial growth is not replicated in synchronized infection of bone marrow-derived macrophages *in vitro*, and further analyses into cytosolic serpin-dependent bacterial growth *in vivo* will be of interest.

Similar to the cGAS-STING cytosolic surveillance pathway, activation of the inflammasome and secretion of IL-1β are dependent on ESX-1 in *M. tuberculosis* and *M. marinum* infection (8, 10, 11). It has been reported that the addition of CA074-Me to *M. tuberculosis* and *M. marinum*-infected macrophages prevents caspase-1 activation and the secretion of IL-1β (40, 41), suggesting a possible role for cathepsin B in inflammasome activation. We find that CA074-Me treatment inhibits ESX-dependent IL-1β secretion, but our genetic analyses demonstrate that the upregulation of cytosolic serpins in *M. marinum* infection regulates cathepsin B activity without affecting inflammasome activation. In this context, it is noteworthy that ESX-1-dependent cell death in *M. marinum*-infected macrophages is independent of inflammasome activation and pyroptosis (8).

Members of the serpin superfamily are upregulated in mice and humans during both bacterial (22, 26, 57–59) and viral (34, 60, 61) infections. While little has been known about their function during infection, it was recently demonstrated that the gene product of *seprina3g* (Spi2A) plays a key role in regulating the inflammatory response in sepsis (59). Clade A3 serpins in mice consist of 14 paralogues to the human acute phase protein α_1_-antichymotrypsin (*SERPINA3*), which is an inhibitor of serine proteases such as cathepsin G (27, 28). These A3 serpins exhibit a high degree of primary sequence conservation, but the reactive center loop—dictating target protease specificity—is highly variable, and the steady-state expression of different A3 serpins may be tissue-specific (30). We find that *M. marinum*-infected macrophages upregulate *serpina3f* and *serpina3g*. Both gene products are cytosolic proteins, and Spi2A is known to bind and inhibit the cysteine protease cathepsin B as it reaches this compartment after leakage from lysosomes (32–39). Lysosomal proteases are normally active at low pH, as in the lysosome, but cathepsin B is active also at neutral pH (44) and its release into the cytosol has been shown to cause cell death (40, 43). The ability of Spi2A to inhibit extralysosomal cathepsin B is cytoprotective during erythropoiesis (37) and in several types of immune cells, including memory CD8 T cells (34, 36) and progenitors of granulocytes and B cells (38), and here, we demonstrate that the gene products of *serpina3f* and/or *serpina3g* contribute to limit cell death of infected macrophages. It will be of interest to evaluate the role of this cytoprotective function during infection with *M. tuberculosis* and other major human pathogens with membranolytic activity.

## MATERIALS AND METHODS

### Animals

Wild-type C57BL/6 (B6) mice and *ifnar1^−/−^* (IFNAR-KO) on the B6 background were bred and maintained at the animal facility at the Department of Biology, Lund University. Frozen B6(FVB)-Serpina3g^tm1.1Arp^/J embryos with an expected *serpina3g*^+/−^ genotype were purchased from the Jackson Laboratory (stock#: 022524) and inserted into female B6. Offspring was genotyped as described below and crossed to generate a stable line of homozygous *serpina3g*^−/−^ (*serpina3g*-KO) mice on the B6 background.

### Genotyping of mice

Genomic DNA was extracted from ear biopsies. Biopsies were incubated for 45 minutes at 95°C in 200 µL NaOH (25 mM) to digest the tissue. Samples were vortexed and neutralized with 35 µL Tris HCl (0.5 M, pH 8.0), and DNA was subsequently separated from digested tissue by centrifugation (3,440 rcf, 5 minutes). Purified DNA was digested with XhoI (Merck, NEBR0146S) for 12 hours at 37°C before heat-inactivation of the restriction enzyme (65°C, 20 minutes). The *serpina3g* genotype was determined by PCR using primers (indicated in Table S1) generating 230 bp and 800 bp fragments for the *serpina3g* deleted and intact alleles, respectively.

### Generation of bone marrow-derived macrophages

Bone marrow-derived mouse macrophages were prepared as previously described (62). In brief, bone marrow cells were flushed from dissected femurs and tibias from B6, IFNAR-KO, and *serpina3g*-KO mice, and cultured for 7 days in macrophage growth medium (RPMI with 10% heat-inactivated fetal bovine serum [FBS; Sigma-Aldrich], 10% 3T3 m-CSF, and 1% glutamine [Thermo Fisher]) for 7 days at 37°C with 5% CO_2_.

### Bacterial strains and growth conditions

Wild-type (WT) *M. marinum* M-strain, an isogenic deletion mutant (∆RD1) lacking the RD1 locus, and ∆RD1 complemented with RD1-2F9 (∆RD1::RD1) have been described previously (10, 63). Bacteria were grown at 30°C in Middlebrook 7H9 medium (BD Biosciences) supplemented with 0.5% glycerol (Sigma-Aldrich), 0.05% Tween 80 (Sigma-Aldrich), and 10% ADC supplement (BD Biosciences), or on Middlebrook 7H10 agar (BD Biosciences) supplemented with 0.5% glycerol and 10% OADC (Conda Lab). Cultures with WT and ∆RD1::RD1 were supplemented with 50 µg/mL hygromycin B (Invitrogen), and ∆RD1 cultures with 50 µg/mL kanamycin (Sigma-Aldrich). For growth on solid media, bacteria were plated on Middlebrook 7H10 agar (BD Biosciences) supplemented with 0.5% glycerol (Sigma-Aldrich) and 10% OADC enrichment (Conda Lab).

### Infection of macrophages

Macrophage infection with *M. marinum* was performed as previously described in detail (62). Briefly, bacteria were washed twice in phosphate-buffered saline (PBS; 2,500 × *g*, 10 minutes), needled three times through a 26G needle, and centrifuged twice (450 × *g*, 1 minute) to achieve a single cell suspension. The bacterial concentration of the suspension was determined using a hemocytometer (KOVA), and macrophages were infected with the appropriate number of bacteria suspended in macrophage medium to obtain the desired multiplicity of infection, and incubated at 32°C with 5% CO_2_. Two hours post-infection, the cells were washed with macrophage medium to remove extracellular bacteria, and the remaining extracellular bacteria were killed off by incubating the cells with macrophage medium supplemented with 200 µg/mL amikacin (Sigma-Aldrich) for 2 hours at 32°C with 5% CO_2_. Cells were then washed twice with macrophage medium and finally incubated in fresh macrophage medium at 32°C with 5% CO_2_ until further analysis. For infections less than 7 hours, the bacteria-containing medium was kept throughout.

### Reverse-transcription quantitative PCR analysis of gene expression

Macrophages were infected in 12-well plates (5 × 10^5^ cells/well), and RNA was extracted at the indicated time post-infection. RNA was isolated using the RNeasy Mini Kit (Qiagen), and cDNA was synthesized using the GoScript Reverse Transcription System (Promega). Gene expression was determined by real-time quantitative PCR using SSoFast EvaGreen qPCR supermix (Bio-Rad) in either 96-well format for the iQ5 Real-Time PCR Detection System (Bio-Rad) or in 384-well format for the CFX384 Touch Real-Time PCR Detection System (Bio-Rad). Expression of the housekeeping gene *reep5* was analyzed to enable normalization of data. All reagents and primers used are indicated in Table S1.

### Analysis of cathepsin B activity

Macrophages were infected in six-well plates (10^6^ cells/well). At the indicated time post-infection, supernatants were collected and centrifuged (300 × *g*, 4 minutes at 4°C) to pellet any detached cells. The purified supernatant fraction was then collected for subsequent analysis of cathepsin B activity. In parallel, macrophages were dislodged by adding 1 mL ice-cold DPBS and pooled with cells collected from the supernatant. The cell suspension (total volume: 1 mL) was then divided into two samples. One sample (700 µL) was used to isolate the cytosolic fraction. For this purpose, the cells were pelleted by centrifugation (1,000 × *g*, 3 minutes at 4°C) and resuspended in 75 µL digitonin buffer (25 µg/mL digitonin [Sigma-Aldrich], 150 mM NaCl, and 50 mM HEPES in PBS) and incubated for 10 minutes on ice, allowing selective permeabilization of the plasma membrane. Permeabilized macrophages were then pelleted by centrifugation (1,000 × *g*, 3 minutes at 4°C), and the supernatant (i.e., the cytosolic fraction) was transferred to a new tube that once again was centrifuged (20,000 × *g*, 3 minutes at 4°C) to pellet any remaining cellular debris. The second sample (300 µL) was pelleted (300 × *g*, 4 minutes at 4°C) and resuspended in 75 µL Triton X-100 buffer (0.1% Triton X-100 [Sigma-Aldrich], 150 mM NaCl, and 50 mM HEPES in PBS) to cause complete lysis of all cellular compartments. The lysate was then centrifuged (20,000 × *g*, 3 minutes at 4°C) to pellet cellular debris, and the supernatant was collected as the whole cell lysate.

To analyze the cathepsin B activity in the prepared fractions (i.e., supernatant, cytosol, and whole cell lysate), 15 µL of each fraction was transferred, in triplicates, to a Nunc 96F untreated black microwell plate (ThermoFisher Scientific). Next, 90 µL of cell free buffer pH 5.5 (10 μM HEPES, 2 mM NaCl, 2.5 mM KH_2_PO_4_, 4 mM EDTA, 2 mM MgCl_2_, and 5 mM pyruvate) was mixed with 1 mM dithiothreitol and 80 µM of the cathepsin B substrate Z-RR-AMC (Sigma-Aldrich), and added to each well. After a 1-hour incubation at 37°C, the fluorescence intensity was measured using Varioskan Lux (ThermoFisher Scientific) at an excitation wavelength of 380 nm and an emitting wavelength of 460 nm. Samples from uninfected macrophages were used as controls.

### Analysis of cytokine secretion and cathepsin B protein concentration

To evaluate secreted protein levels, macrophages were infected in 12-well plates (5 × 10^5^ cells/well). At 24 hpi, supernatants were collected and centrifuged (300 × *g*, 5 minutes at 4°C) to pellet any detached cells. The supernatants were then analyzed by enzyme-linked immunosorbent assay (ELISA) for IL-6 (eBioscience), IFNβ (RnD Systems), and IL-1β (RnD Systems), according to manufacturers’ instructions. To determine the concentration of cathepsin B protein in the cytosol of infected macrophages, the cytosolic fraction was isolated, as described above and analyzed by ELISA for cathepsin B (Abcam), according to the manufacturer’s instructions.

### Intracellular growth of *M. marinum*

For analysis of intracellular growth, macrophages were infected in 96-well plates (5 × 10^4^ cells/well) and lysed with 0.1% Triton X-100 (Sigma-Aldrich) for 10 minutes at room temperature at the indicated time points post-infection. Tenfold serial dilutions of the lysates were plated on 7H10 agar plates for CFU analysis.

### Analysis of LDH release

Macrophages were seeded to 96-well plates (5 × 10^4^ cells/well). Supernatants were collected at the indicated time point and centrifuged (300 × *g*, 5 minutes at 4°C) to pellet any detached cells. The supernatant was subsequently analyzed for LDH using the colorimetric CytoTox 96 Non-Radioactive Cytotoxicity Assay (Promega), according to the manufacturer’s instructions.

### Infection of mice

Female mice between 8 and 12 weeks of age were infected with *M. marinum* (200 µL, 7 × 10^7^ CFU/mL in PBS) intravenously through the tail vein, as previously described in detail (62, 64). Disease development was analyzed every other day by measuring the length of all visible skin lesions on the tail of each mouse. At indicated time points, tails and tail-draining lymph nodes (sciatic and inguinal) were harvested for CFU analysis. Lymph nodes were collected in PBS supplemented with 0.1% Triton X-100 and homogenized by bead beating using TissueLyser II (Qiagen). Tails were severed from mice at the tail base, cut into 3 mm pieces, and homogenized in PBS supplemented with 0.1% Triton X-100 using homogenizer PT 1200 E (Polytron) as described (62, 64). Tissue lysates were serially diluted and plated on 7H10 agar plates for enumeration of CFUs.

### RNA extraction from infected tail tissue

Tails were severed from mice at the tail base, and RNA was purified as previously described (64). The tissue was separated from the bone after a longitudinal excision and immediately stored at −80°C. Frozen tissue was immersed in liquid nitrogen and pulverized with a biopulverizer (Biospec Products) that had been prechilled in liquid nitrogen. Pulverized tissue samples were resuspended in 0.5 mL PBS and homogenized with a homogenizer PT 1200 E (Polytron). RNA was extracted from the homogenized tissue using the RNeasy Mini Kit (Qiagen) with DNase digestion. Gene expression analysis was performed as described above.

### Flow cytometry analysis of infected tail tissue

Flow cytometry analysis was performed on single cell suspensions prepared from infected tail tissue as previously described in detail (64). In brief, tails were severed from mice at the tail base, and the separated tissue was cut into small pieces that were incubated in DMEM supplemented with 5% fetal calf serum (FCS), 30  µg/mL Liberase (Roche), and 52  µg/mL DNase I (Sigma) with magnetic stirring for 60 minutes at 37°C. Samples were passed through a 70-µm nylon cell strainer, washed in PBS supplemented with 3% FCS and 2 mM EDTA, and filtered through a 40-µm nylon cell strainer to obtain single cell suspensions. Cells were incubated with a rat anti-mouse CD16/CD32 antibody (clone 2.4G2) to block Fc-receptors, and subsequently stained (30 minutes, on ice) with the fixable viable dye Near-IR Dead Cell Stain Kit (Invitrogen) and the following fluorochrome-conjugated anti-mouse antibodies in PBS: CD45.2 (clone 104), CD11b (clone M1/70), CD64 (clone X54-5/7.1), Ly6C (clone HK1.4), Ly6G (clone 1A8), MHCII (clone M5/114.15.2), CD19 (clone 6D5), TCRβ (clone H57-597), CD3 (clone 17A2). Cells were fixed with 2% paraformaldehyde for 20 minutes at room temperature. Each sample was supplemented with AccCount Fluorescent Particles (Spherotech) to enable the determination of the total cell count. Flow cytometry analysis was conducted using an LSR II flow cytometer (BD Sciences), and data were processed using the FlowJo software version 10.

### Statistical analysis

Statistical analysis was performed using the software GraphPad Prism version 8. A one-way analysis of variance (ANOVA) with Tukey’s test or a two-way ANOVA with Tukey’s test were used for multiple comparisons, as indicated in figure legends. A two-tailed unpaired *t*-test was used for pairwise comparisons. A *P* < 0.05 was considered significant; *P < 0.05, **P < 0.01, ***P < 0.001, and ****P < 0.0001.

## SUPPLEMENTAL MATERIAL

Fig. S1 mbio.00384-24-s0001.tif Expression of A3 serpins at 24 hpi.

Fig. S2 mbio.00384-24-s0002.tif Cytosolic serpins do not affect ESX-1-dependent production of type I IFN in *M. marinum*-infected macrophages.

Fig. S3 mbio.00384-24-s0003.tif The cytoprotective effect of cytosolic serpins is overridden at 24 hpi *in vitro*.

Fig. S4 mbio.00384-24-s0004.tif Mycobacteria grow comparably in B6 and serpina3g-KO macrophages *in vitro*.

Fig. S5 mbio.00384-24-s0005.tif Lack of cytosolic serpins does not affect the cellularity of neutrophils and C64+ cells in the infected tissue.

Legends mbio.00384-24-s0006.docx Legends for supplemental material.

Table S1 mbio.00384-24-s0007.pdf Materials and reagents.

ASM does not own the copyrights to Supplemental Material that may be linked to, or accessed through, an article. The authors have granted ASM a non-exclusive, world-wide license to publish the Supplemental Material files. Please contact the corresponding author directly for reuse.

## References

[1] Stinear TP, Seemann T, Harrison PF, Jenkin GA, Davies JK, Johnson PDR, Abdellah Z, Arrowsmith C, Chillingworth T, Churcher C, et al.. 2008. Insights from the complete genome sequence of Mycobacterium marinum on the evolution of Mycobacterium tuberculosis. Genome Res 18:729–741. doi:10.1101/gr.075069.107 18403782 PMC2336800

[2] Wolf AJ, Linas B, Trevejo-Nuñez GJ, Kincaid E, Tamura T, Takatsu K, Ernst JD. 2007. Mycobacterium tuberculosis infects dendritic cells with high frequency and impairs their function in vivo. J Immunol 179:2509–2519. doi:10.4049/jimmunol.179.4.2509 17675513

[3] Lienard J, Munke K, Wulff L, Da Silva C, Vandamme J, Laschanzky K, Joeris T, Agace W, Carlsson F. 2023. Intragranuloma accumulation and inflammatory differentiation of neutrophils underlie mycobacterial ESX-1-dependent immunopathology. mBio 14:e0276422. doi:10.1128/mbio.02764-22 37017530 PMC10127687

[4] Cohen SB, Gern BH, Delahaye JL, Adams KN, Plumlee CR, Winkler JK, Sherman DR, Gerner MY, Urdahl KB. 2018. Alveolar macrophages provide an early Mycobacterium tuberculosis niche and initiate dissemination. Cell Host Microbe 24:439–446. doi:10.1016/j.chom.2018.08.001 30146391 PMC6152889

[5] Hsu T, Hingley-Wilson SM, Chen B, Chen M, Dai AZ, Morin PM, Marks CB, Padiyar J, Goulding C, Gingery M, Eisenberg D, Russell RG, Derrick SC, Collins FM, Morris SL, King CH, Jacobs WR. 2003. The primary mechanism of attenuation of bacillus Calmette-Guérin is a loss of secreted lytic function required for invasion of lung interstitial tissue. Proc Natl Acad Sci U S A 100:12420–12425. doi:10.1073/pnas.1635213100 14557547 PMC218773

[6] Stanley SA, Raghavan S, Hwang WW, Cox JS. 2003. Acute infection and macrophage subversion by Mycobacterium tuberculosis require a specialized secretion system. Proc Natl Acad Sci U S A 100:13001–13006. doi:10.1073/pnas.2235593100 14557536 PMC240734

[7] Volkman HE, Clay H, Beery D, Chang JCW, Sherman DR, Ramakrishnan L. 2004. Tuberculous granuloma formation is enhanced by a mycobacterium virulence determinant. PLoS Biol 2:e367. doi:10.1371/journal.pbio.0020367 15510227 PMC524251

[8] Carlsson F, Kim J, Dumitru C, Barck KH, Carano RAD, Sun M, Diehl L, Brown EJ. 2010. Host-detrimental role of Esx-1-mediated inflammasome activation in mycobacterial infection. PLoS Pathog 6:e1000895. doi:10.1371/journal.ppat.1000895 20463815 PMC2865529

[9] Gao LY, Guo S, McLaughlin B, Morisaki H, Engel JN, Brown EJ. 2004. A mycobacterial virulence gene cluster extending RD1 is required for cytolysis, bacterial spreading and ESAT-6 secretion. Mol Microbiol 53:1677–1693. doi:10.1111/j.1365-2958.2004.04261.x 15341647

[10] Koo IC, Wang C, Raghavan S, Morisaki JH, Cox JS, Brown EJ. 2008. ESX-1-dependent cytolysis in lysosome secretion and inflammasome activation during mycobacterial infection. Cell Microbiol 10:1866–1878. doi:10.1111/j.1462-5822.2008.01177.x 18503637 PMC2574867

[11] Kurenuma T, Kawamura I, Hara H, Uchiyama R, Daim S, Dewamitta SR, Sakai S, Tsuchiya K, Nomura T, Mitsuyama M. 2009. The RD1 locus in the Mycobacterium tuberculosis genome contributes to activation of caspase-1 via induction of potassium ion efflux in infected macrophages. Infect Immun 77:3992–4001. doi:10.1128/IAI.00015-09 19596775 PMC2737993

[12] Lienard J, Nobs E, Lovins V, Movert E, Valfridsson C, Carlsson F. 2020. The Mycobacterium marinum ESX-1 system mediates phagosomal permeabilization and type I interferon production via separable mechanisms. Proc Natl Acad Sci U S A 117:1160–1166. doi:10.1073/pnas.1911646117 31879349 PMC6969537

[13] Watson RO, Manzanillo PS, Cox JS. 2012. Extracellular M. tuberculosis DNA targets bacteria for autophagy by activating the host DNA-sensing pathway. Cell 150:803–815. doi:10.1016/j.cell.2012.06.040 22901810 PMC3708656

[14] Manzanillo PS, Shiloh MU, Portnoy DA, Cox JS. 2012. Mycobacterium tuberculosis activates the DNA-dependent cytosolic surveillance pathway within macrophages. Cell Host Microbe 11:469–480. doi:10.1016/j.chom.2012.03.007 22607800 PMC3662372

[15] Collins AC, Cai H, Li T, Franco LH, Li X-D, Nair VR, Scharn CR, Stamm CE, Levine B, Chen ZJ, Shiloh MU. 2015. Cyclic GMP-AMP synthase is an innate immune DNA sensor for Mycobacterium tuberculosis. Cell Host Microbe 17:820–828. doi:10.1016/j.chom.2015.05.005 26048137 PMC4499468

[16] Wassermann R, Gulen MF, Sala C, Perin SG, Lou Y, Rybniker J, Schmid-Burgk JL, Schmidt T, Hornung V, Cole ST, Ablasser A. 2015. Mycobacterium tuberculosis differentially activates cGAS- and inflammasome-dependent intracellular immune responses through ESX-1. Cell Host Microbe 17:799–810. doi:10.1016/j.chom.2015.05.003 26048138

[17] Watson RO, Bell SL, MacDuff DA, Kimmey JM, Diner EJ, Olivas J, Vance RE, Stallings CL, Virgin HW, Cox JS. 2015. The cytosolic sensor cGAS detects Mycobacterium tuberculosis DNA to induce type I interferons and activate autophagy. Cell Host Microbe 17:811–819. doi:10.1016/j.chom.2015.05.004 26048136 PMC4466081

[18] Movert E, Lienard J, Valfridsson C, Nordström T, Johansson-Lindbom B, Carlsson F. 2018. Streptococcal M protein promotes IL-10 production by cGAS-independent activation of the STING signaling pathway. PLoS Pathog 14:e1006969. doi:10.1371/journal.ppat.1006969 29579113 PMC5886698

[19] Wiens KE, Ernst JD. 2016. The mechanism for type I interferon induction by Mycobacterium tuberculosis is bacterial strain-dependent. PLoS Pathog 12:e1005809. doi:10.1371/journal.ppat.1005809 27500737 PMC4976988

[20] Berry MPR, Graham CM, McNab FW, Xu Z, Bloch SAA, Oni T, Wilkinson KA, Banchereau R, Skinner J, Wilkinson RJ, Quinn C, Blankenship D, Dhawan R, Cush JJ, Mejias A, Ramilo O, Kon OM, Pascual V, Banchereau J, Chaussabel D, O’Garra A. 2010. An interferon-inducible neutrophil-driven blood transcriptional signature in human tuberculosis. Nature 466:973–977. doi:10.1038/nature09247 20725040 PMC3492754

[21] Manca C, Tsenova L, Bergtold A, Freeman S, Tovey M, Musser JM, Barry CE, Freedman VH, Kaplan G. 2001. Virulence of a Mycobacterium tuberculosis clinical isolate in mice is determined by failure to induce Th1 type immunity and is associated with induction of IFN-α/β. Proc Natl Acad Sci U S A 98:5752–5757. doi:10.1073/pnas.091096998 11320211 PMC33285

[22] Stanley SA, Johndrow JE, Manzanillo P, Cox JS. 2007. The type I IFN response to infection with Mycobacterium tuberculosis requires ESX-1-mediated secretion and contributes to pathogenesis. J Immunol 178:3143–3152. doi:10.4049/jimmunol.178.5.3143 17312162

[23] McNab FW, Ewbank J, Rajsbaum R, Stavropoulos E, Martirosyan A, Redford PS, Wu X, Graham CM, Saraiva M, Tsichlis P, Chaussabel D, Ley SC, O’Garra A. 2013. TPL-2–ERK1/2 signaling promotes host resistance against intracellular bacterial infection by negative regulation of type I IFN production. J Immunol 191:1732–1743. doi:10.4049/jimmunol.1300146 23842752 PMC3796877

[24] Dorhoi A, Yeremeev V, Nouailles G, Weiner J, Jörg S, Heinemann E, Oberbeck-Müller D, Knaul JK, Vogelzang A, Reece ST, Hahnke K, Mollenkopf H-J, Brinkmann V, Kaufmann SHE. 2014. Type I IFN signaling triggers immunopathology in tuberculosis-susceptible mice by modulating lung phagocyte dynamics. Eur J Immunol 44:2380–2393. doi:10.1002/eji.201344219 24782112 PMC4298793

[25] Moreira-Teixeira L, Mayer-Barber K, Sher A, O’Garra A. 2018. Type I interferons in tuberculosis: foe and occasionally friend. J Exp Med 215:1273–1285. doi:10.1084/jem.20180325 29666166 PMC5940272

[26] Hamerman JA, Hayashi F, Schroeder LA, Gygi SP, Haas AL, Hampson L, Coughlin P, Aebersold R, Aderem A. 2002. Serpin 2a is induced in activated macrophages and conjugates to a ubiquitin homolog. J Immunol 168:2415–2423. doi:10.4049/jimmunol.168.5.2415 11859133

[27] Forsyth S, Horvath A, Coughlin P. 2003. A review and comparison of the murine a1-antitrypsin and a1-antichymotrypsin multigene clusters with the human clade A serpins. Genomics 81:336–345. doi:10.1016/s0888-7543(02)00041-1 12659817

[28] de Mezer M, Rogaliński J, Przewoźny S, Chojnicki M, Niepolski L, Sobieska M, Przystańska A. 2023. SERPINA3: stimulator or inhibitor of pathological changes. Biomedicines 11:156. doi:10.3390/biomedicines11010156 36672665 PMC9856089

[29] Morris EC, Dafforn TR, Forsyth SL, Missen MA, Horvath AJ, Hampson L, Hampson IN, Currie G, Carrell RW, Coughlin PB. 2003. Murine serpin 2A is a redox-sensitive intracellular protein. Biochem J 371:165–173. doi:10.1042/BJ20021567 12470299 PMC1223254

[30] Horvath AJ, Forsyth SL, Coughlin PB. 2004. Expression patterns of murine antichymotrypsin-like genes reflect evolutionary divergence at the serpina3 locus. J Mol Evol 59:488–497. doi:10.1007/s00239-004-2640-9 15638460

[31] Heit C, Jackson BC, McAndrews M, Wright MW, Thompson DC, Silverman GA, Nebert DW, Vasiliou V. 2013. Update of the human and mouse SERPIN gene superfamily. Hum Genomics 7:22. doi:10.1186/1479-7364-7-22 24172014 PMC3880077

[32] Liu N, Raja SM, Zazzeroni F, Metkar SS, Shah R, Zhang M, Wang Y, Brömme D, Russin WA, Lee JC, Peter ME, Froelich CJ, Franzoso G, Ashton-Rickardt PG. 2003. NF-kappaB protects from the lysosomal pathway of cell death. EMBO J 22:5313–5322. doi:10.1093/emboj/cdg510 14517268 PMC204493

[33] Liu N, Wang Y, Ashton-Rickardt PG. 2004. Serine protease inhibitor 2A inhibits caspase-independent cell death. FEBS Lett 569:49–53. doi:10.1016/j.febslet.2004.05.061 15225607

[34] Liu N, Phillips T, Zhang M, Wang Y, Opferman JT, Shah R, Ashton-Rickardt PG. 2004. Serine protease inhibitor 2A is a protective factor for memory T cell development. Nat Immunol 5:919–926. doi:10.1038/ni1107 15311278

[35] Kreuzaler PA, Staniszewska AD, Li W, Omidvar N, Kedjouar B, Turkson J, Poli V, Flavell RA, Clarkson RWE, Watson CJ. 2011. Stat3 controls lysosomal-mediated cell death in vivo. Nat Cell Biol 13:303–309. doi:10.1038/ncb2171 21336304

[36] Byrne SM, Aucher A, Alyahya S, Elder M, Olson ST, Davis DM, Ashton-Rickardt PG. 2012. Cathepsin B controls the persistence of memory CD8+ T lymphocytes. J Immunol 189:1133–1143. doi:10.4049/jimmunol.1003406 22745374 PMC3401340

[37] Dev A, Byrne SM, Verma R, Ashton-Rickardt PG, Wojchowski DM. 2013. Erythropoietin-directed erythropoiesis depends on serpin inhibition of erythroblast lysosomal cathepsins. J Exp Med 210:225–232. doi:10.1084/jem.20121762 23319700 PMC3570101

[38] Li L, Byrne SM, Rainville N, Su S, Jachimowicz E, Aucher A, Davis DM, Ashton-Rickardt PG, Wojchowski DM. 2014. Brief report: serpin Spi2A as a novel modulator of hematopoietic progenitor cell formation. Stem Cells 32:2550–2556. doi:10.1002/stem.1778 24964278 PMC4138266

[39] Shamji MH, Temblay JN, Cheng W, Byrne SM, Macfarlane E, Switzer AR, Francisco NDC, Olexandra F, Jacubczik F, Durham SR, Ashton-Rickardt PG. 2018. Antiapoptotic serine protease inhibitors contribute to survival of allergenic TH2 cells. J Allergy Clin Immunol 142:569–581. doi:10.1016/j.jaci.2017.07.055 29106998 PMC5920800

[40] Abdallah AM, Bestebroer J, Savage NDL, de Punder K, van Zon M, Wilson L, Korbee CJ, van der Sar AM, Ottenhoff THM, van der Wel NN, Bitter W, Peters PJ. 2011. Mycobacterial secretion systems ESX-1 and ESX-5 play distinct roles in host cell death and inflammasome activation. J Immunol 187:4744–4753. doi:10.4049/jimmunol.1101457 21957139

[41] Amaral EP, Riteau N, Moayeri M, Maier N, Mayer-Barber KD, Pereira RM, Lage SL, Kubler A, Bishai WR, D’Império-Lima MR, Sher A, Andrade BB. 2018. Lysosomal cathepsin release is required for NLRP3-inflammasome activation by Mycobacterium tuberculosis in infected macrophages. Front Immunol 9:1427. doi:10.3389/fimmu.2018.01427 29977244 PMC6021483

[42] Zhang G, Wang J, Zhao Z, Xin T, Fan X, Shen Q, Raheem A, Lee CR, Jiang H, Ding J. 2022. Regulated necrosis, a proinflammatory cell death, potentially counteracts pathogenic infections. Cell Death Dis 13:637. doi:10.1038/s41419-022-05066-3 35869043 PMC9307826

[43] Lee J, Remold HG, Ieong MH, Kornfeld H. 2006. Macrophage apoptosis in response to high intracellular burden of Mycobacterium tuberculosis is mediated by a novel caspase-independent pathway. J Immunol 176:4267–4274. doi:10.4049/jimmunol.176.7.4267 16547264

[44] Yoon MC, Solania A, Jiang Z, Christy MP, Podvin S, Mosier C, Lietz CB, Ito G, Gerwick WH, Wolan DW, Hook G, O’Donoghue AJ, Hook V. 2021. Selective neutral pH inhibitor of cathepsin B designed based on cleavage preferences at cytosolic and lysosomal pH conditions. ACS Chem Biol 16:1628–1643. doi:10.1021/acschembio.1c00138 34416110 PMC9104225

[45] Amaral EP, Costa DL, Namasivayam S, Riteau N, Kamenyeva O, Mittereder L, Mayer-Barber KD, Andrade BB, Sher A. 2019. A major role for ferroptosis in Mycobacterium tuberculosis-induced cell death and tissue necrosis. J Exp Med 216:556–570. doi:10.1084/jem.20181776 30787033 PMC6400546

[46] Wong KW, Jacobs WR. 2011. Critical role for NLRP3 in necrotic death triggered by Mycobacterium tuberculosis. Cell Microbiol 13:1371–1384. doi:10.1111/j.1462-5822.2011.01625.x 21740493 PMC3257557

[47] Roca FJ, Whitworth LJ, Redmond S, Jones AA, Ramakrishnan L. 2019. TNF induces pathogenic programmed macrophage necrosis in tuberculosis through a mitochondrial-lysosomal-endoplasmic reticulum circuit. Cell 178:1344–1361. doi:10.1016/j.cell.2019.08.004 31474371 PMC6736209

[48] Zhang L, Jiang X, Pfau D, Ling Y, Nathan CF. 2021. Type I interferon signaling mediates Mycobacterium tuberculosis-induced macrophage death. J Exp Med 218:e20200887. doi:10.1084/jem.20200887 33125053 PMC7608065

[49] van der Wel N, Hava D, Houben D, Fluitsma D, van Zon M, Pierson J, Brenner M, Peters PJ. 2007. M. tuberculosis and M. leprae translocate from the phagolysosome to the cytosol in myeloid cells. Cell 129:1287–1298. doi:10.1016/j.cell.2007.05.059 17604718

[50] Simeone R, Bobard A, Lippmann J, Bitter W, Majlessi L, Brosch R, Enninga J. 2012. Phagosomal rupture by Mycobacterium tuberculosis results in toxicity and host cell death. PLoS Pathog 8:e1002507. doi:10.1371/journal.ppat.1002507 22319448 PMC3271072

[51] Teles RMB, Graeber TG, Krutzik SR, Montoya D, Schenk M, Lee DJ, Komisopoulou E, Kelly-Scumpia K, Chun R, Iyer SS, Sarno EN, Rea TH, Hewison M, Adams JS, Popper SJ, Relman DA, Stenger S, Bloom BR, Cheng G, Modlin RL. 2013. Type I interferon suppresses type II interferon-triggered human anti-mycobacterial responses. Science 339:1448–1453. doi:10.1126/science.1233665 23449998 PMC3653587

[52] Lienard J, Movert E, Valfridsson C, Sturegård E, Carlsson F. 2016. ESX-1 exploits type I IFN-signalling to promote a regulatory macrophage phenotype refractory to IFNγ-mediated autophagy and growth restriction of intracellular mycobacteria. Cell Microbiol 18:1471–1485. doi:10.1111/cmi.12594 27062290

[53] Novikov A, Cardone M, Thompson R, Shenderov K, Kirschman KD, Mayer-Barber KD, Myers TG, Rabin RL, Trinchieri G, Sher A, Feng CG. 2011. Mycobacterium tuberculosis triggers host type I IFN signaling to regulate IL-1b production in human macrophages. J Immunol 187:2540–2547. doi:10.4049/jimmunol.1100926 21784976 PMC3159798

[54] Mayer-Barber KD, Andrade BB, Barber DL, Hieny S, Feng CG, Caspar P, Oland S, Gordon S, Sher A. 2011. Innate and adaptive interferons suppress IL-1α and IL-1β production by distinct pulmonary myeloid subsets during Mycobacterium tuberculosis infection. Immunity 35:1023–1034. doi:10.1016/j.immuni.2011.12.002 22195750 PMC3246221

[55] Mayer-Barber KD, Andrade BB, Oland SD, Amaral EP, Barber DL, Gonzales J, Derrick SC, Shi R, Kumar NP, Wei W, Yuan X, Zhang G, Cai Y, Babu S, Catalfamo M, Salazar AM, Via LE, Barry CE, Sher A. 2014. Host-directed therapy of tuberculosis based on interleukin-1 and type I interferon crosstalk. Nature 511:99–103. doi:10.1038/nature13489 24990750 PMC4809146

[56] Norris BA, Ernst JD. 2018. Mononuclear cell dynamics in M. tuberculosis infection provide opportunities for therapeutic intervention. PLoS Pathog 14:e1007154. doi:10.1371/journal.ppat.1007154 30365557 PMC6221360

[57] Reece ST, Loddenkemper C, Askew DJ, Zedler U, Schommer-Leitner S, Stein M, Mir FA, Dorhoi A, Mollenkopf H-J, Silverman GA, Kaufmann SHE. 2010. Serine protease activity contributes to control of Mycobacterium tuberculosis in hypoxic lung granulomas in mice. J Clin Invest 120:3365–3376. doi:10.1172/JCI42796 20679732 PMC2929725

[58] Toossi Z, Wu M, Rojas R, Kalsdorf B, Aung H, Hirsch CS, Walrath J, Wolbink A, van Ham M, Silver RF. 2012. Induction of serine protease inhibitor 9 by Mycobacterium tuberculosis inhibits apoptosis and promotes survival of infected macrophages. J Infect Dis 205:144–151. doi:10.1093/infdis/jir697 22090449 PMC3242743

[59] Wang X, Ding Y, Li R, Zhang R, Ge X, Gao R, Wang M, Huang Y, Zhang F, Zhao B, Liao W, Du J. 2023. N6-methyladenosine of Spi2a attenuates inflammation and sepsis-associated myocardial dysfunction in mice. Nat Commun 14:1185. doi:10.1038/s41467-023-36865-7 36864027 PMC9979126

[60] Suvarna K, Biswas D, Pai MGJ, Acharjee A, Bankar R, Palanivel V, Salkar A, Verma A, Mukherjee A, Choudhury M, Ghantasala S, Ghosh S, Singh A, Banerjee A, Badaya A, Bihani S, Loya G, Mantri K, Burli A, Roy J, Srivastava A, Agrawal S, Shrivastav O, Shastri J, Srivastava S. 2021. Proteomics and machine learning approaches reveal a set of prognostic markers for COVID-19 severity with drug repurposing potential. Front Physiol 12:652799. doi:10.3389/fphys.2021.652799 33995121 PMC8120435

[61] Nuñez E, Orera I, Carmona-Rodríguez L, Paño JR, Vázquez J, Corrales FJ. 2022. Mapping the serum proteome of COVID-19 patients; guidance for severity assessment. Biomedicines 10:1690. doi:10.3390/biomedicines10071690 35884998 PMC9313396

[62] Lienard J, Carlsson F. 2017. Murine Mycobacterium marinum infection as a model for tuberculosis. Methods Mol Biol 1535:301–315. doi:10.1007/978-1-4939-6673-8_20 27914088

[63] Cosma CL, Humbert O, Ramakrishnan L. 2004. Superinfecting mycobacteria home to established tuberculous granulomas. Nat Immunol 5:828–835. doi:10.1038/ni1091 15220915

[64] Lienard J, Munke K, Carlsson F. 2023. A murine Mycobacterium marinum infection model for longitudinal analyses of disease development and the inflammatory response. Methods Mol Biol 2674:313–326. doi:10.1007/978-1-0716-3243-7_21 37258977

