# Supplementary figures and images for: Cytosolic serpins act in a cytoprotective feedback loop that limits ESX-1-dependent death of Mycobacterium marinum-infected macrophages
Source: mBio. 2024 Aug 1;15(9):e00384-24. doi: 10.1128/mbio.00384-24 (PMC11389378; doi:10.1128/mbio.00384-24)

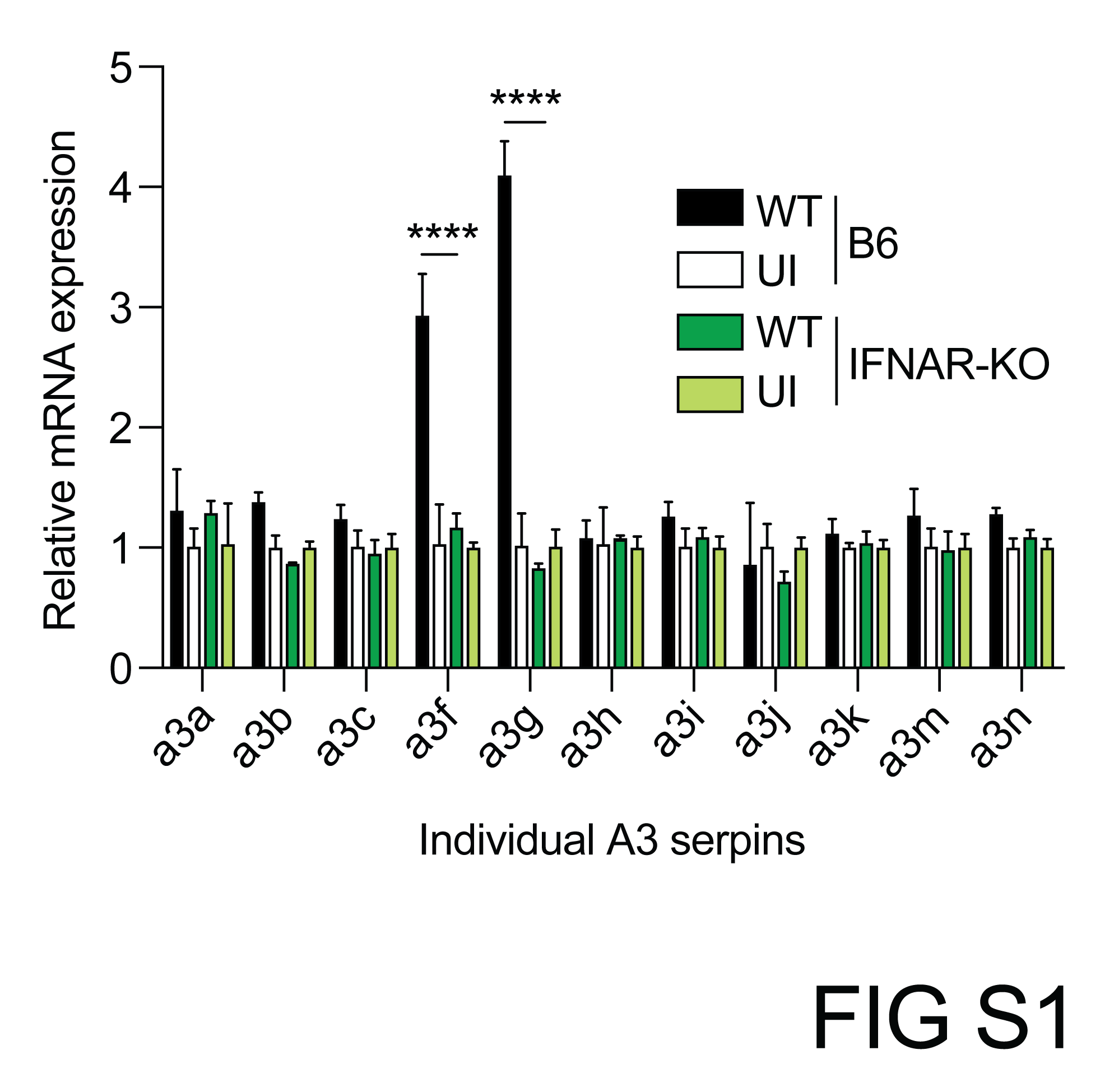

Supplement: Fig. S1 — Expression of A3 serpins at 24 hpi. [file mbio.00384-24-s0001.tif]

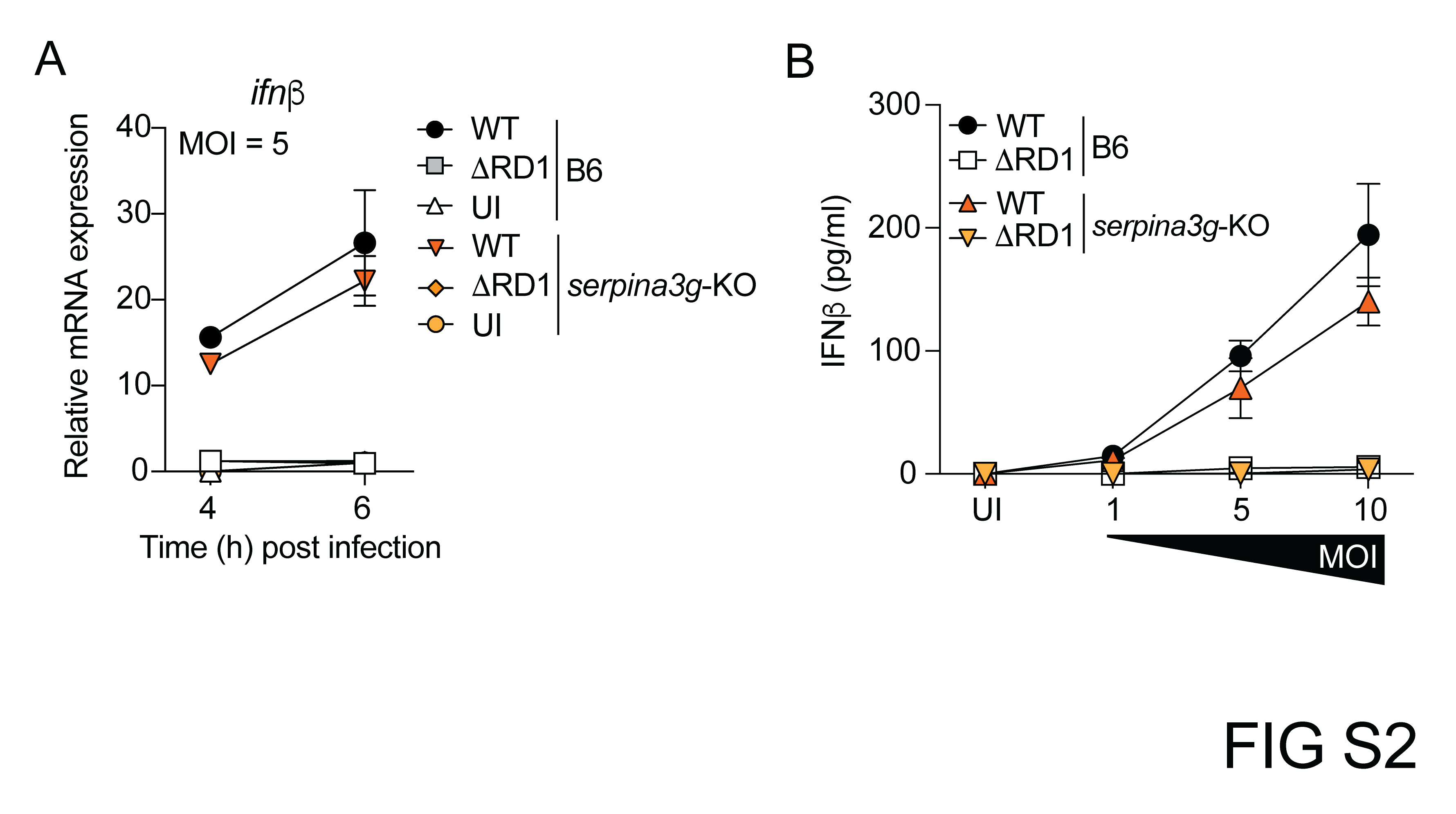

Supplement: Fig. S2 — Cytosolic serpins do not affect ESX-1-dependent production of type I IFN in M. marinum-infected macrophages. [file mbio.00384-24-s0002.tif]

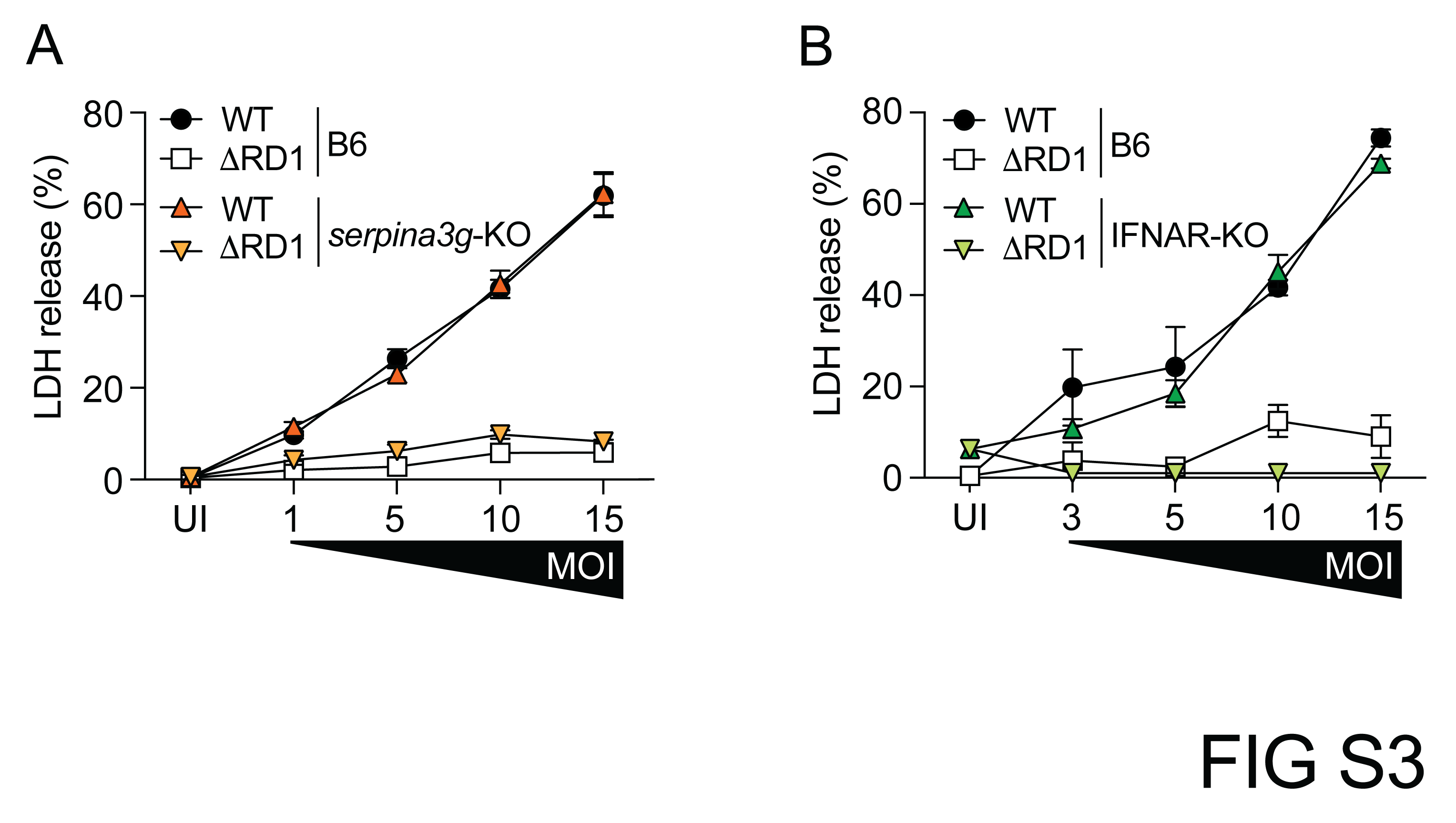

Supplement: Fig. S3 — The cytoprotective effect of cytosolic serpins is overridden at 24 hpi in vitro. [file mbio.00384-24-s0003.tif]

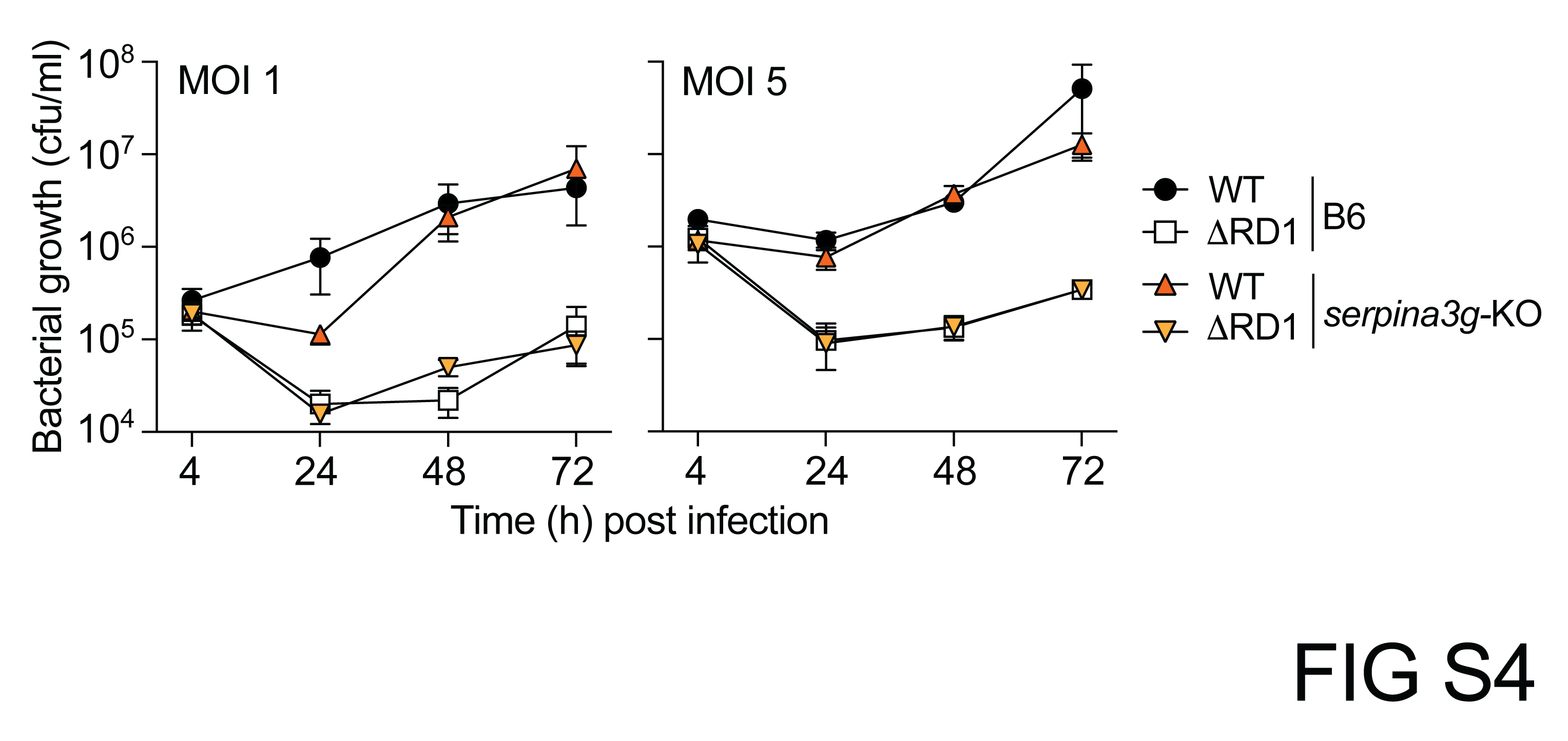

Supplement: Fig. S4 — Mycobacteria grow comparably in B6 and serpina3g-KO macrophages in vitro. [file mbio.00384-24-s0004.tif]

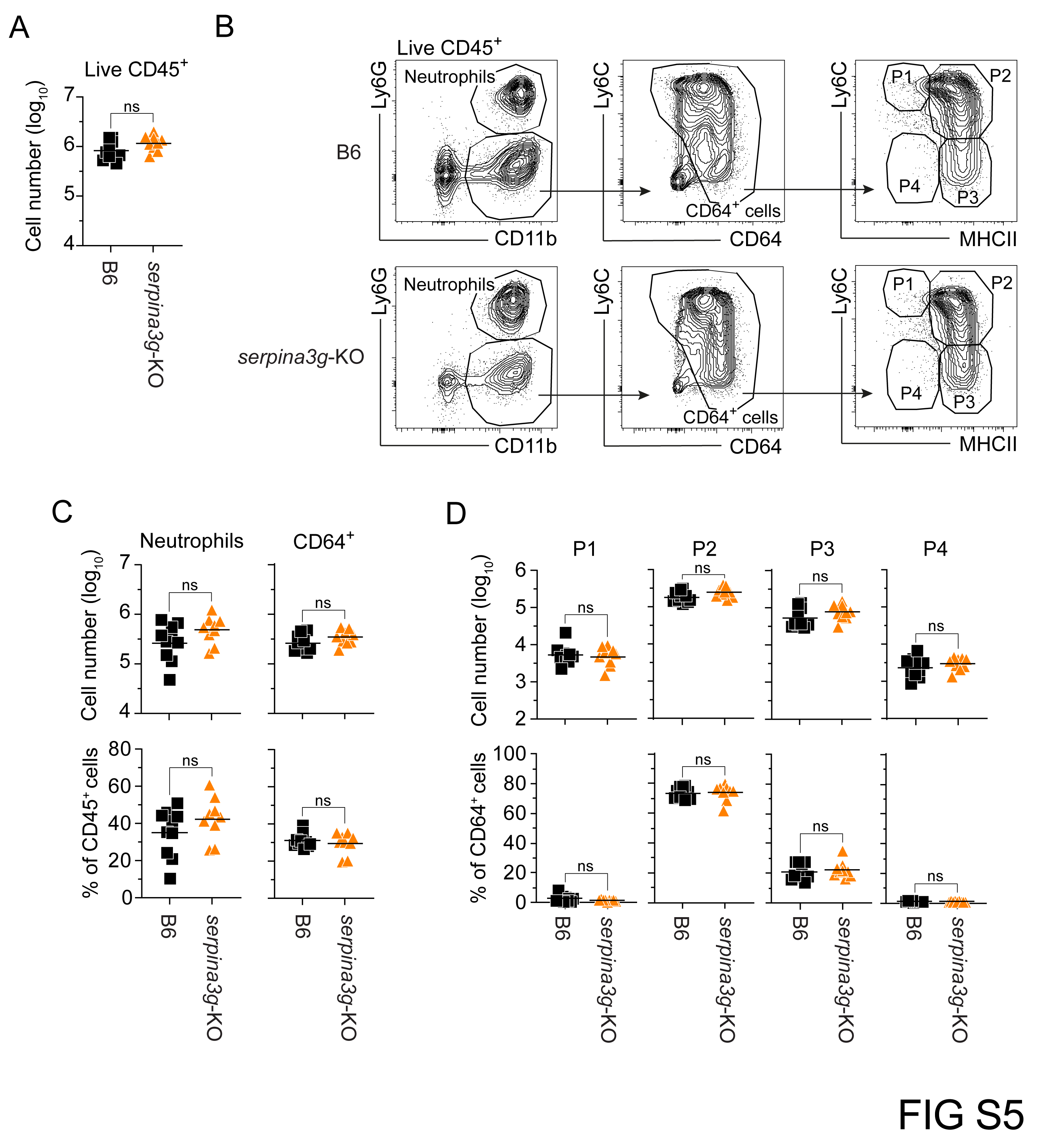

Supplement: Fig. S5 — Lack of cytosolic serpins does not affect the cellularity of neutrophils and C64+ cells in the infected tissue. [file mbio.00384-24-s0005.tif]
