# Supplementary material for: Cytosolic serpins act in a cytoprotective feedback loop that limits ESX-1-dependent death of Mycobacterium marinum-infected macrophages
Source: mBio. 2024 Aug 1;15(9):e00384-24. doi: 10.1128/mbio.00384-24 (PMC11389378; doi:10.1128/mbio.00384-24)
Supplement: Legends — for supplemental material. [file mbio.00384-24-s0006.docx]

**SUPPLEMENTAL MATERIAL CAPTIONS**

**FIG S1 Expression of A3 serpins at 24 hpi**. B6 and IFNAR-KO macrophages were infected with WT and ΔRD1 *M. marinum* (MOI 5), or UI as indicated. Expression of members of the A3 serpins were investigated by RTqPCR at 24 hpi. Results are represented as fold-change relative to UI control. Results (mean and SD; *n* = 3) are representative of 3 independent experiments (2-way ANOVA, ****p<0.0001).

**FIG S2 Cytosolic serpins do not affect ESX-1-dependent production of type I IFN in *M. marinum* infected macrophages.** B6 and *serpina3g*-KO macrophages were infected with WT and ΔRD1 *M. marinum*, or uninfected (UI), as indicated. **A)** Expression of *ifnβ* was analyzed by RTqPCR at the indicated timepoints post infection. **B)** The concentration of secreted IFNβ was determined by ELISA at 24 hpi. **(A, B)** Results (mean ± SD; *n* = 3) are representative of 3 independent experiments (2-way ANOVA, no statistical difference).

**FIG S3 The cytoprotective effect of cytosolic serpins is overridden at 24 hpi *in vitro*. A and B)** B6, IFNAR-KO and *serpina3g*-KO macrophages were infected with WT or ΔRD *M. marinum*, or UI as indicated. Lactate dehydrogenase (LDH)-release was measured at 24 hpi at titrated MOIs, as indicated. Results (mean ± SD; *n* = 3) are representative of 3 independent experiments (2-way ANOVA, no statistical difference).

**FIG S4 Mycobacteria grow comparably in B6 and *serpina3g*-KO macrophages *in vitro*.** B6 and *serpina3g*-KO macrophages were infected with WT or ΔRD1 *M. marinum* at MOI = 1 and 5, as indicated. Intracellular bacterial growth was analyzed at the indicated time points. Results (mean ± SD; *n* = 3) are representative of 3 independent experiments (2-way ANOVA, no statistical difference).

**FIG S5 Lack of cytosolic serpins does not affect the cellularity of neutrophils and C64^+^ cells in the infected tissue.** Flow cytometry analysis of tail tissues from B6 and *serpina3g*-KO mice infected with WT *M. marinum* (1.4 x 10^7^ CFUs). Analysis performed at 14 days post infection. **A)** Number of hematopoietic cells (CD45^+^). **B)** Gating strategy defining neutrophils (Ly6G^+^CD11b^+^), monocyte-derived cells and macrophages (CD64^+^), as well as the subdivision of the CD64^+^ compartment into gates 1 to 4 (P1 to P4) based on MHCII and Ly6C expression. Cells in the left panels are lineage-negative (CD3-, TCRβ- and CD19-negative) live CD45^+^ cells. **C)** Quantification of the number (top panels) and relative abundance (as percent of CD45^+^ cells; lower panels) of neutrophils and CD64^+^ cells, as defined in panel B. **D)** Quantification of the number (top panels) and relative abundance (as percent of CD64^+^ cells) of cells within the P1 to P4 gates, as defined in panel B. Results (*n* = 9-10 mice per group) from two independent experiments. Bars indicate the mean for each group. Two-tailed unpaired t test, no statistical difference (ns).

**TABLE S1** Materials and reagents.
