# Supplementary material for: Cytosolic serpins act in a cytoprotective feedback loop that limits ESX-1-dependent death of Mycobacterium marinum-infected macrophages
Source: mBio. 2024 Aug 1;15(9):e00384-24. doi: 10.1128/mbio.00384-24 (PMC11389378; doi:10.1128/mbio.00384-24)
Supplement: Table S1 — Materials and reagents. [file mbio.00384-24-s0007.pdf]

TABLE S1. Materials and reagents.

***Mycobacterium marinum* strains**	**Reference**	
Wild type (WT)	Volkman *et al.* PLoS Biol 2004	
∆RD1	Volkman *et al.* PLoS Biol 2004	
∆RD1::RD1-2F9	Koo *et al.* Cell Microbiol 2008	
**Commercial Assays**	**Source**	**Catalog number**
Mouse IFNβ ELISA kit	RnD Systems	Cat# DY8234-05
Mouse IL1β ELISA kit	RnD Systems	Cat# DY401-05
Mouse IL-6 ELISA kit	eBioscience	Cat# 29-806160
Mouse cathepsin B ELISA kit	Abcam	Cat# ab119585
CytoTox96® Non-Radioactive Cytotoxicity Assay	Promega	Cat# G1780
RNeasy® Mini Kit	Qiagen	Cat# 74106
GoScript™ Reverse Transcription System	Promega	Cat# A5001
SSoFast EvaGreen qPCR supermix	Bio-Rad	Cat# 1725204
RNase-Free DNase Set	Qiagen	Cat# 79245
LIVE/DEAD™ Fixable Near-IR Dead Cell Stain Kit	Thermo Fischer	Cat# L34975
**Chemicals**	**Source**	**Catalog number**
Hygromycin B	Invitrogen	Cat# 10687010
Kanamycin Sulfate	Sigma-Aldrich	Cat# 60615
Middlebrook 7H9	BD Biosciences	Cat# 271310
ADC enrichment for 7H9	BD Biosciences	Cat# 212352
Middlebrook 7H10	BD Biosciences	Cat# 262710
OADC enrichment for 7H10	Conda Lab	Cat# 6037
Tween 80	Sigma-Aldrich	Cat# P1754
Glycerol	Sigma-Aldrich	Cat# G6279
RPMI-1640	Gibco™/LifeTechnologies	Cat# 31870-025
Fetal Bovine Serum Lot 074M3264	Sigma-Aldrich	Cat# F7524
Amikacin	Sigma-Aldrich	Cat# A3650
L-Glutamine	Gibco™/LifeTechnologies	Cat# 25030-024
Digitonin	Sigma-Aldrich	Cat# D141
Triton X-100	ThermoFisher Scientific	Cat# T8787
XhoI	Merck	Cat# NEBR0146S
Hot Start Taq kit	Qiagen	Cat# 203203
Z-RR-AMC	Sigma-Aldrich	Cat# C5429
Liberase TM	Roche	Cat# 05401127001
DNAse I	Sigma-Aldrich	Cat# 10104159001
Paraformaldehyde	Alfa Aesar/Thermo Fisher	Cat# J61899

AccCount Fluorescent Particles	Spherotech	Cat# ACFP-70-10
**Inhibitors**	**Source**	**Catalog number**
CA074-Me	CalBiochem	Cat# 205531
E64	Sigma-Aldrich	Cat# E3132
Pepstatin A	Sigma-Aldrich	Cat# P5318
RKLLW-NH2	Sigma-Aldrich	Cat# SCP0110
**Primers**	**Sequence (5' - 3')**	
reep5 (forward)	GATACCCAGCCTACATCTCAATG	
reep5 (reverse)	GCAATGCTGAACACACCATATAC	
ifnb (forward)	ATGAGTGGTGGTTGCAGGC	
ifnb (reverse)	TGACCTTTCAAATGCAGTAGATTCA	
cathepsin B (forward)	TCCTTGATCCTTCTTTCTTGCC	
cathepsin B (reverse)	ACAGTGCCACACAGCTTCTTC	
a3 serpins (forward)	TTTCCAGCAACCTCTCAAGGC	
a3 serpins (reverse)	CTGGGTGTGATTGCCCACATA	
serpina3a (forward)	AGATGTCATCACAATAGCCCG	
serpina3a (reverse)	TTTGTTAAAGGGTTGATAACC	
serpina3b (forward)	GATGCAATCACAATAGTCGGATAC	
serpina3b (reverse)	GGTTGATAACCTTGCCCATTAC	
serpina3c (forward)	CTTAGTAGAAGAACCAGTCTG	
serpina3c (reverse)	CTTAGGGTGAGTGATTTTGGC	
serpina3f (forward)	ATCTCCAATGTTGTCAAGGTG	
serpina3f (reverse)	TGAAACTTTTGCCATAAAGAG	
serpina3g (forward)	ACAGGAATGGCAGGTGTCGG	
serpina3g (reverse)	TGTAACTTTTGCCATAAAGA	
serpina3h (forward)	ACAGGGGTCAAATTAATTC	
serpina3h (reverse)	CTTGGGATTTGTAACTTTGGC	
serpina3i (forward)	AGGAGTCAAAGTTAATCTACG	
serpina3i (reverse)	CTTGGGATTTGTAACTTTGGC	
serpina3j (forward)	CAAGAGACAAATATGACTTC	
serpina3j (reverse)	GTGCAGGGTTGTTGATCTTGC	
serpina3k (forward)	GGCCATTCCTGATTGTTATC	
serpina3k (reverse)	TGAGAACTTGGTGAGCTTTA	
serpina3m (forward)	GCTTTCGTTCTAGAAGATTAC	
serpina3m (reverse)	CTTGGGGTTAGTGACTTTGGC	
serpina3n (forward)	CAATGTCTGCGAAACTGTACC	
serpina3n (reverse)	TTTGGGGTTGGCTATCTTGGC	
**Primers for genotyping**	**Sequence (5' - 3')**	
oSB073	GGGCGGTGTAGGCCGCTGGACC	
oSB080	CCATCTGGGCATGTGGGATTGGGAC	
oSB085	CCAGAAAGTCAAAAACTGCACAACATCCG	
oSB086	CTGGAGCACATCCTTCCTGAGTTGG	
Antibodies	Source	Catalog number
Monoclonal rat anti-CD3	BioLegend	Cat# 100227, clone 17A2
Monoclonal rat anti-CD11b	BioLegend	Cat# 101237, clone M1/70
Monoclonal rat anti-CD19	BioLegend	Cat# 115537, clone 6D5
Monoclonal mouse anti-CD45.2	eBiosciences	Cat# 56-0454-82, clone 104

Monoclonal mouse anti-CD64	BD Biosciences	Cat# 558539, clone X54-5/7.1
Monoclonal rat anti-Ly6C	BioLegend	Cat# 128012, clone HK1.4
Monoclonal rat anti-Ly6G	BD Biosciences	Cat# 563979, clone 1A8
Monoclonal rat anti-MHCII/I-A/I-E	BD Biosciences	Cat# 563415, clone M5/114.15.2
Monoclonal Armenian Hamster anti-TCRβ	BioLegend	Cat# 109230, clone H57-597
Purified rat anti-CD16/CD32 block	BD Biosciences	Cat# 553142, clone 2.4G2
**Equipment**	**Source**	
iQ5 Real-Time PCR Detection System	Bio-Rad	
CFX384 Touch Real-Time PCR Detection System	Bio-Rad	
SPECTROstar	BMG Labtech	
Varioskan Lux	ThermoFisher Scientific	
TissueLyser II	Qiagen	
Biopulverizer	Biospec Products	
Homogenizer PT1200 E	Polytron	
Flow cytometer LSRII	BD Biosciences	
**Software**	**Source**	
Prism 9	GraphPad	
Illustrator 2023	Adobe	
CFX Maestro	Bio-Rad	
FlowJo, version 9.9.6 and v10.7.1	BD Biosciences	
FACSDiva, version 8.0	BD Biosciences	
